# Seven confluence principles: a case study of standardized statistical analysis for 26 methods that assign net atomic charges in molecules

**DOI:** 10.1039/d0ra06392d

**Published:** 2020-12-15

**Authors:** Thomas A. Manz

**Affiliations:** Chemical & Materials Engineering, New Mexico State University Las Cruces New Mexico 88003-3805 USA tmanz@nmsu.edu

## Abstract

This article studies two kinds of information extracted from statistical correlations between methods for assigning net atomic charges (NACs) in molecules. First, relative charge transfer magnitudes are quantified by performing instant least squares fitting (ILSF) on the NACs reported by Cho *et al.* (*ChemPhysChem*, 2020, **21**, 688–696) across 26 methods applied to ∼2000 molecules. The Hirshfeld and Voronoi deformation density (VDD) methods had the smallest charge transfer magnitudes, while the quantum theory of atoms in molecules (QTAIM) method had the largest charge transfer magnitude. Methods optimized to reproduce the molecular dipole moment (*e.g.*, ACP, ADCH, CM5) have smaller charge transfer magnitudes than methods optimized to reproduce the molecular electrostatic potential (*e.g.*, CHELPG, HLY, MK, RESP). Several methods had charge transfer magnitudes even larger than the electrostatic potential fitting group. Second, confluence between different charge assignment methods is quantified to identify which charge assignment method produces the best NAC values for predicting *via* linear correlations the results of 20 charge assignment methods having a complete basis set limit across the dataset of ∼2000 molecules. The DDEC6 NACs were the best such predictor of the entire dataset. Seven confluence principles are introduced explaining why confluent quantitative descriptors offer predictive advantages for modeling a broad range of physical properties and target applications. These confluence principles can be applied in various fields of scientific inquiry. A theory is derived showing confluence is better revealed by standardized statistical analysis (*e.g.*, principal components analysis of the correlation matrix and standardized reversible linear regression) than by unstandardized statistical analysis. These confluence principles were used together with other key principles and the scientific method to make assigning atom-in-material properties non-arbitrary. The N@C_60_ system provides an unambiguous and non-arbitrary falsifiable test of atomic population analysis methods. The HLY, ISA, MK, and RESP methods failed for this material.

## Abstract 2

Standardized statistical analysis of many methods to assign net atomic charges revealed relative charge transfer magnitudes and confluent correlations.

## Graphical abstract

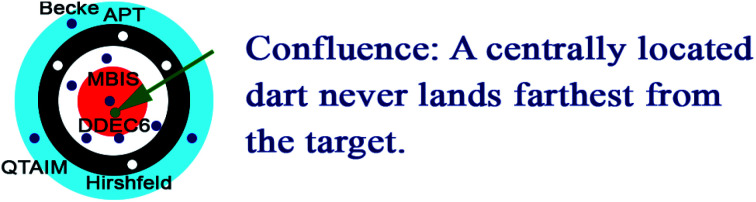

## 1. Introduction

Herein, statistical analysis is performed to better understand relationships among the large number of different methods for assigning net atomic charges (NACs) to atoms in molecules. Two related topics are explored. First, how do the relative charge transfer magnitudes of different NAC methods compare? Which NAC methods exhibit relatively small charge transfer magnitudes compared to other methods? Which exhibit relatively large charge transfer magnitudes? Second, which NAC method should be selected if the goal is to model a diverse set of properties related to NACs? For example, which NAC method assigns NACs having the overall strongest linear correlations to various other methods for assigning NACs?

Answering these questions requires an extensive dataset for statistical analysis. Cho *et al.* computed NACs for ∼2000 molecules and ions using 26 different charge assignment methods.^1^ These charge assignment methods spanned many categories, including: (a) electron density partitioning into overlapping atoms, (b) electron density partitioning into non-overlapping atoms, (c) NACs optimized to reproduce the molecular electrostatic potential (MEP), molecular dipole moment, or molecular dipole moment derivatives, (d) projection of the first-order density matrix to give NACs having a complete basis set limit, (e) projection of the first-order density matrix to give NACs having no complete basis set limit, and (f) various other schemes. The ∼2000 systems they studied were from the GMTKN55 database, which includes main group molecules and ions.^2^ Cho *et al.*'s quantum chemistry calculations were performed using the PBE0 hybrid functional,^3,4^ def2-TZVPP basis set,^5^ and using geometries from the online GMTKN55 database^2^ without further optimization. Their dataset comprises 29 934 atoms-in-molecules for which NACs were reported.^1^

The present article studies the general question of how to design computed quantitative descriptors that are correlated to experimentally observed measured properties, where the computed quantitative descriptor itself is not unambiguously measurable experimentally for most materials. For most materials, the charge of an atom in the material is not itself unambiguously measurable experimentally.^6^ Nevertheless, centuries of chemical science history show regarding some atoms in materials as positively charged (aka cations) and others as negatively charged (aka anions) is extremely useful for conceptually explaining chemical properties of materials.^7^ Therefore, NAC is a useful computed quantitative descriptor for modeling or explaining experimentally observable properties such as molecular dipole moments, electric field surrounding molecule, chemical reactivity, spectroscopic properties, *etc.* that are related to atom-in-material charges.

Is it possible to make any definite statements about how strongly correlated different NAC definitions are to any conceivable experimentally measured chemical property related to atom-in-material charges simply by studying statistical correlations in-between different NAC definitions even without knowing the experimentally measured chemical property to be modeled or explained? Surprisingly, I show herein the answer is yes. I derive a theory of confluence that shows some definitions for assigning NACs are positioned to produce average or better correlations to any and all conceivable properties related to atom-in-material charges. By the same reasoning, a bond order definition can be constructed that exhibits average or better correlations to any and all conceivable chemical properties related to bond orders. Accordingly, assigning properties to atoms in materials is not arbitrary.

More generally, this theory of confluence has transformative implications for all mathematical and physical sciences wherever the goal is to design a computed quantitative descriptor that is itself not a direct experimental observable (at least in most cases) but is correlated to a large number of experimentally observable properties. Confluence means a “joining together”. Here, I show many statistical properties that were formerly considered distinct have strict equivalence or near-equivalence that eliminates much of the ambiguity in statistical analyses. Specifically, the seven confluence principles explained herein show how to design a broadly applicable quantitative descriptor that exhibits average or better correlations to any and all conceivable related properties. Much like the theory of quantum mechanics that was developed in the twentieth century, this theory of confluence has profound and wide-ranging impacts that force us to interpret the world around us in new ways. This theory of confluence shows that defining quantitative descriptors that are not unambiguously measurable experimentally is still not an arbitrary process, because statistical correlations in-between possible alternative definitions determine which definition exhibits average or better correlations to any and all conceivable related properties.

The rest of this article is organized as follows. Section 2 explains the computational methods and theory behind them. Section 2.1 describes how the source data was checked for consistency to remove a small number of bad data points. Section 2.2 describes the rational and procedure for using a standardized reversible least squares fitting called instant least squares fitting (ILSF) to compute the relative charge transfer magnitudes of different charge assignment methods. Section 2.3 describes the principal components analysis (PCA) method. Section 2.4 presents mathematical theory governing maximally correlated descriptors. Section 3 presents computational results. Section 3.1 uses ILSF to quantify charge transfer magnitudes and explains atomic population method classification. Section 3.2 identifies highly correlated descriptors using the correlation matrix and PCA applied to the NAC database. Section 3.3 presents results on the sensitivity of ranking to the choice of included charge assignment methods. Section 3.4 compares computed AIM populations for a benchmark system having unambiguous experimental values. Section 4 explains seven confluence principles that comprise the theory of confluence. Section 5 explains how these confluence principles work together with other key principles and the scientific method to make assigning atom-in-material properties non-arbitrary. Section 6 concludes. Section 7 contains several mathematical proofs.

## 2. Methods

### 2.1 Checking the source data for consistency

I checked the source NAC database^1^ for consistency as follows. Because the correct net charge of every molecule or ion in the database is integer-valued, the running sum of NACs should reach an integer for the last atom-in-material of every molecule or ion in the database. The database was divided into blocks containing approximately 500 atoms-in-materials per block. Each block contained many molecules/ions, and each molecule/ion belonged to only one block. (A system containing two molecules or ions spaced far apart (aka ‘spatially separated’) could be divided into two blocks, with one whole molecule or ion in each block.) For each charge assignment method, the running sum of NACs was computed for each block. For a particular block, the running sum should be equivalent between any two charge assignment methods.

Discrepancies between this expected behavior took three forms. First, some of the methods that computed NACs by numerical real-space integration had small, negligible integration errors; these NACs required no correction. Second, the MBSBickelhaupt NACs were missing for an extremely small number of atoms in materials. This occurred for a spatially separated Li^+^ ion in four places, for which the MBSBickelhaupt NAC was manually set to +1. A [Li·(OH_2_)]^+^ complex was missing MBSBickelhaupt NACs, so this system was entirely removed from the dataset for all charge assignment methods. Third, erroneous quantum theory of atoms in molecules (QTAIM) NACs were reported for a few systems. The spatially separated Li_2_ (two occurrences), B_2_, C_2_, and P_2_ (three occurrences) QTAIM NACs were manually set to zero, because they were erroneously reported to have large NACs (+0.26 to +0.65). Two systems containing 7 (*i.e.*, H_3_Li_3_C) and 16 (*i.e.*, H_7_BO_2_NaMg_2_Al_2_Cl) atoms were removed from all charge assignment methods, because their erroneously reported QTAIM NACs did not approximately sum to the system's net charge.

These corrections reduced the number of atoms in materials in the dataset from 29 934 to 29 907. After these corrections, the running sums were approximately consistent for all charge assignment methods. Because these corrections affected an extremely small percentage (∼0.1%) of the dataset, the overall statistical behaviors of the dataset were negligibly impacted by these corrections. The corrected dataset containing 29 907 atoms in materials was used for all statistical analysis reported here. The charge assignment methods in this dataset included: atomic charge partitioning (ACP),^8^ atomic dipole corrected Hirshfeld (ADCH),^9^ atomic polar tensor (APT),^10^ Becke,^11^ Bickelhaupt,^12^ charges from electrostatic potentials using a grid (CHELPG),^13^ charge model 5 (CM5),^14^ sixth generation density-derived electrostatic and chemical (DDEC6),^15^ electronegativity equilibration (EEQ),^16–20^ Hirshfeld,^21^ intrinsic bond orbital (IBO),^22^ Hu-Lu-Yang electrostatic potential fitting (HLY),^23^ iterative atomic charge partitioning (i-ACP),^24^ iterative Hirshfeld (Hirshfeld-I),^25^ iterated stockholder atoms (ISA),^26^ minimal basis iterative stockholder (MBIS),^27^ minimal basis set Bickelhaupt projection (MBSBickelhaupt),^1^ minimal basis set Mulliken projection (MBSMulliken),^28^ Merz-Kollman electrostatic potential fitting (MK),^29^ Mulliken,^30^ natural population analysis (NPA),^31^ quantum theory of atoms in molecules (QTAIM),^32^ restrained electrostatic potential fitting (RESP),^33^ Ros–Schuit,^34^ Stout–Politzer,^35^ and Voronoi deformation density (VDD).^36^

### 2.2 Instant least squares fitting (ILSF)

Let {*α*_*i*_} and {*β*_*i*_} denote the NAC sets of two methods, where the subscript *i* runs over all atoms in materials. Standard deviations are computed in the usual manner:1
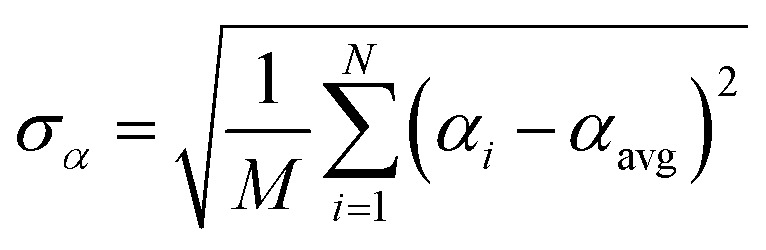
where *M* = (*N* − 1) for a sample standard deviation and *M* = *N* for a population standard deviation.^37^ As described in standard statistics textbooks, the population standard deviation is computed from every datapoint in an entire population, while the sample standard deviation is computed when a data subset has been drawn from a larger population.^37^ All equations in this article work whether the {*σ*} correspond to sample or population standard deviations, but the same choice must be made for all regressed variables. Herein, the entire population of 29 907 atoms in materials were used to compute *σ* (*i.e.*, *M* = *N* = 29 907).

The covariance matrix is defined as^38^2
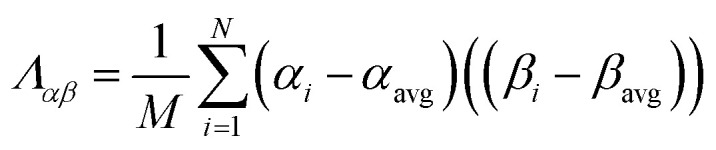
If *Λ*_*αβ*_ = 0, *Λ*_*αβ*_ > 0, or *Λ*_*αβ*_ < 0, the two variables *α* and *β* are said to be uncorrelated, positively correlated, or negatively correlated, respectively.^37^ The covariance of a variable with itself is called that variable's variance:^37^

3 *Λ*_*αα*_ = (*σ*_*α*_)^2^

The correlation matrix is defined as^37,38^

4 −1 ≤ *Ω*_*αβ*_ = *Λ*_*αβ*_/(*σ*_*α*_*σ*_*β*_) ≤ 1

From eqn (4), the covariance and correlation matrices equal each other when all variables have unit standard deviation:

5 *Ω*_*wz*_ = *Λ*_*wz*_ when *σ*_w_ = *σ*_z_ = 1

This can be achieved by standardizing the variables:^39^

6 *w*_*i*_ = *

<svg xmlns="http://www.w3.org/2000/svg" version="1.0" width="14.727273pt" height="16.000000pt" viewBox="0 0 14.727273 16.000000" preserveAspectRatio="xMidYMid meet"><metadata>
Created by potrace 1.16, written by Peter Selinger 2001-2019
</metadata><g transform="translate(1.000000,15.000000) scale(0.015909,-0.015909)" fill="currentColor" stroke="none"><path d="M480 840 l0 -40 -40 0 -40 0 0 -40 0 -40 40 0 40 0 0 40 0 40 40 0 40 0 0 -40 0 -40 40 0 40 0 0 40 0 40 -40 0 -40 0 0 40 0 40 -40 0 -40 0 0 -40z M240 520 l0 -40 -40 0 -40 0 0 -40 0 -40 -40 0 -40 0 0 -160 0 -160 40 0 40 0 0 -40 0 -40 120 0 120 0 0 40 0 40 40 0 40 0 0 40 0 40 40 0 40 0 0 -80 0 -80 80 0 80 0 0 40 0 40 -40 0 -40 0 0 40 0 40 -40 0 -40 0 0 80 0 80 40 0 40 0 0 120 0 120 -40 0 -40 0 0 -40 0 -40 -40 0 -40 0 0 40 0 40 -120 0 -120 0 0 -40z m240 -80 l0 -40 40 0 40 0 0 -40 0 -40 -40 0 -40 0 0 -80 0 -80 -40 0 -40 0 0 -40 0 -40 -120 0 -120 0 0 120 0 120 40 0 40 0 0 40 0 40 40 0 40 0 0 40 0 40 80 0 80 0 0 -40z"/></g></svg>

*_*i*_ = (*α*_*i*_ − *α*_avg_)*s*_*α*_/*σ*_*α*_

7 *z*_*i*_ = *

<svg xmlns="http://www.w3.org/2000/svg" version="1.0" width="11.058824pt" height="16.000000pt" viewBox="0 0 11.058824 16.000000" preserveAspectRatio="xMidYMid meet"><metadata>
Created by potrace 1.16, written by Peter Selinger 2001-2019
</metadata><g transform="translate(1.000000,15.000000) scale(0.010294,-0.010294)" fill="currentColor" stroke="none"><path d="M640 1320 l0 -40 -40 0 -40 0 0 -40 0 -40 40 0 40 0 0 40 0 40 40 0 40 0 0 -40 0 -40 40 0 40 0 0 40 0 40 -40 0 -40 0 0 40 0 40 -40 0 -40 0 0 -40z M480 1080 l0 -40 -40 0 -40 0 0 -40 0 -40 -40 0 -40 0 0 -120 0 -120 -40 0 -40 0 0 -160 0 -160 -40 0 -40 0 0 -120 0 -120 -40 0 -40 0 0 -80 0 -80 40 0 40 0 0 40 0 40 40 0 40 0 0 80 0 80 160 0 160 0 0 40 0 40 40 0 40 0 0 40 0 40 40 0 40 0 0 160 0 160 -40 0 -40 0 0 40 0 40 40 0 40 0 0 40 0 40 40 0 40 0 0 80 0 80 -40 0 -40 0 0 40 0 40 -120 0 -120 0 0 -40z m240 -120 l0 -80 -40 0 -40 0 0 -40 0 -40 -80 0 -80 0 0 -40 0 -40 40 0 40 0 0 -40 0 -40 40 0 40 0 0 -120 0 -120 -80 0 -80 0 0 -40 0 -40 -80 0 -80 0 0 120 0 120 40 0 40 0 0 160 0 160 40 0 40 0 0 80 0 80 120 0 120 0 0 -80z"/></g></svg>

*_*i*_ = (*β*_*i*_ − *β*_avg_)*s*_*β*_/*σ*_*β*_

where

8 (*s*_*α*_)^2^ = (*s*_*β*_)^2^ = 1

Least-squares regression is a potential way to simultaneously quantify the relative charge transfer magnitudes and correlations between two methods for assigning NACs. Linear models could be constructed as

9 *α*_*i*_ ≈ *mβ*_*i*_ + *c* = *α*^pred^_*i*_

10 *β*_*i*_ ≈ *m*′*α*_*i*_ + *c*′ = *β*^pred^_*i*_

If these two models are equivalent, then solving eqn (9) for *β*_*i*_ yields an equation equal to eqn (10). Examining eqn (9) and (10), these two linear models are equivalent if

11 *m*′ = 1/*m* and *c*′ = −*c*/*m*

We define a reversible least-squares fitting as one for which fitting {*α*_*i*_} to {*β*_*i*_} (eqn (9)) yields a model equivalent to fitting {*β*_*i*_} to {*α*_*i*_} (eqn (10)). Because simple least squares fitting minimizes12
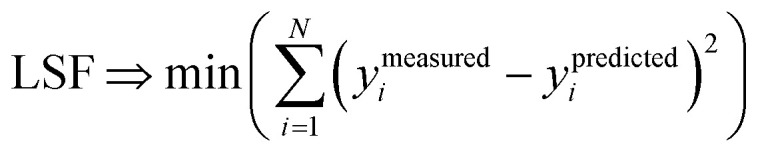
the results of simple least squares fitting of {*α*_*i*_} to {*β*_*i*_} is not equivalent to fitting {*β*_*i*_} to {*α*_*i*_}.

For example, simple least squares fitting yields the two inequivalent models

13 VDD = 0.1641 × QTAIM + 0.0016

14 QTAIM = 3.7470 × VDD − 0.0054

where VDD are the VDD NACs, and QTAIM are the QTAIM NACs. The contradiction between these two models is obvious. Specifically, solving eqn (13) for QTAIM gives QTAIM = 6.0951 × VDD − 0.0099, which does not even approximately equal eqn (14).

**Fig. 1:**
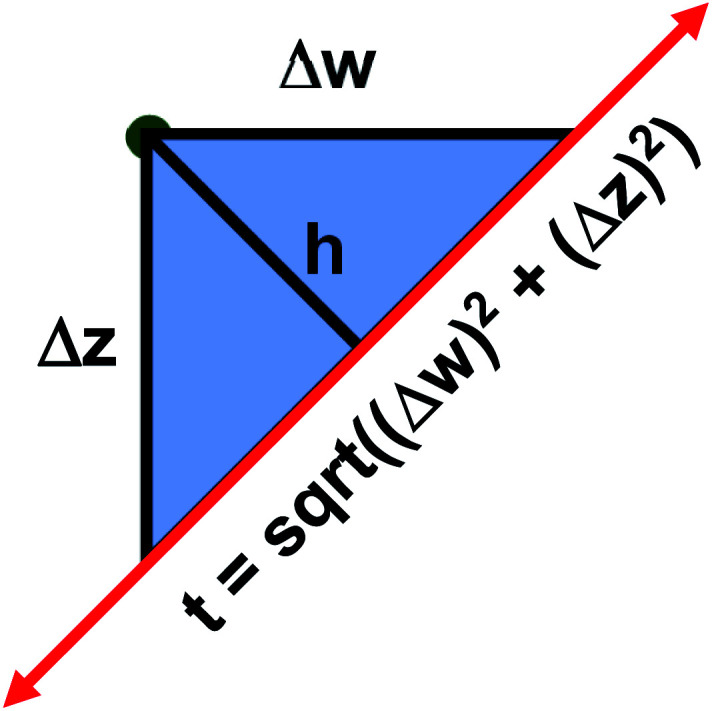
Geometry illustrating the error measures used in total least squares (approach 1) and orthogonal distance regression (approach 2). The red line represents the model equation. The green dot represents the measured datapoint. Approach 1 minimizes *t*^2^, and approach 2 minimizes *h*^2^.

The two approaches illustrated in Fig. 1 solve this problem. Both approaches minimize the squared deviations in both *w* and *z* variables:15
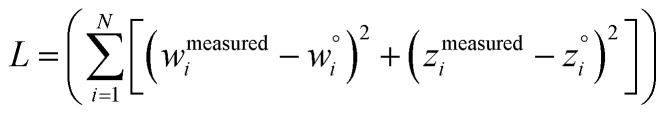


Because eqn (15) is symmetric with respect to swapping the *w* and *z* variables, this is a reversible least squares fitting. The two approaches differ in how 
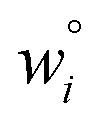
 and 
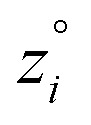
 are chosen. In approach 1 (aka total least squares^40,41^ with a Euclidean metric), 
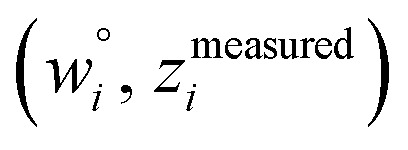
 and 
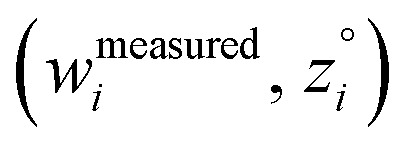
 are horizontally and vertically lined up with (*w*^measured^_*i*_, *z*^measured^_*i*_), respectively. In approach 2 (aka orthogonal distance regression^40–42^), 
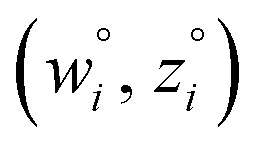
 is the closest point on the model line to (*w*^measured^_*i*_, *z*^measured^_*i*_), and this corresponds to the line between these two points being perpendicular to the model line.

Orthogonal distance regression was shown to be equivalent to a special case of total least squares regression.^40,41^ Moreover, the resulting linear model for orthogonal distance regression corresponds to the major axis in principal components analysis (PCA).^38,41,42^ Here, I show that by standardizing the independent variables it is possible to achieve a quadfecta for bivariate linear regression between any two positively correlated quantitative descriptors. Namely, the simultaneous accomplishment of: (1) orthogonal distance regression, (2) total least squares regression with Euclidean metric, (3) PCA regression, and (4) an instantaneous universal bivariate linear model. I now prove this instant least-squares fitting (ILSF) can be achieved by standardizing the variables (eqn (6)–(7)), where *s*_*α*_ = 1 and *s*_*β*_ = *sign*(*Λ*_*αβ*_). If *Λ*_*αβ*_ = 0, then *w*_*i*_ and *z*_*i*_ are uncorrelated, and the model collapses to the point (*α*^model^_*i*_, *β*^model^_*i*_)=(*α*_avg_, *β*_avg_). Otherwise, *w*_*i*_ and *z*_*i*_ are positively correlated and the ILSF yields the extremely simple linear model

16 *α̂*^model^_*i*_ = *β̂*^model^_*i*_

A remarkable property of eqn (16) is this linear model equation is identical for all conceivable pairs (*α̂*_*i*_, *β̂*_*i*_) of positively correlated real-valued standardized variables. That is, the same model equation describes the ILSF between any conceivable pair of real-valued positively correlated standardized quantitative descriptors in the universe. The name ‘instant least squares fitting’ denotes the amazing result that the ILSF optimized linear model of eqn (16) can be written down instantaneously without having to perform computerized calculations. Section 7.1 below proves this ILSF model simultaneously optimizes the total least squares and orthogonal distance regression of the standardized variables.

ILSF is not the same as Deming regression. In Deming regression, deviations in the *x* and *y* variables are normalized by their measurement uncertainties (which approximately equal their root-mean-squared deviations from the model line).^42,43^ In ILSF, standardized variables are used which normalize deviations in the *x* and *y* variables by the root-mean-squared deviations from their average values. Also, ILSF is not the same as a simple least-squares fit on two standardized variables, because simple least squares fitting yields irreversible models.

### 2.3 Principal components analysis (PCA)

PCA finds the eigenvalues and eigenvectors of the correlation and/or covariance matrices.^38,39,44^ The principal components are sorted from highest to lowest eigenvalue.^38,39,44^ The eigenvector having the largest eigenvalue is the first (aka ‘main’) principal component.^38,39,44^

The PCA eigenvectors are uncorrelated to each other (*i.e.*, the covariance between any two different eigenvectors is zero).^38,39,44^ This naturally follows from the fact that eigenvectors of any real symmetric matrix can be represented as an orthonormal basis.^38,45^ If no eigenvalue is repeated (*i.e.*, all eigenvalues are distinct), then the orthonormal eigenvectors are uniquely determined.^45^ However, if two or more eigenvalues are equal, any rotation of the subspace formed from the corresponding eigenvectors yields new (and equally good) eigenvectors having the same eigenvalue.^38,45^

For standardized variables, the correlation and covariance matrices are equal yielding unique results. For unstandardized variables, PCA of the correlation matrix is invariant to rescaling the variables, while PCA of the covariance matrix is not.^38^ For example, consider PCA of three variables (*A*, *B*, *C*) compared to PCA of (*A*, *B*, *D*) where *D* is defined as 2*C*. PCA of the correlation matrix yields identical results for both variable sets, while PCA of the covariance matrix does not.

For PCA of the covariance matrix, the main principal component is the linear combination

17 *P*_*i*_^(*k*)^ = *C*^(*k*,*j*)^*X*_*i*_^(*j*)^

that results in the highest possible variance, subject to the normalization constraint18
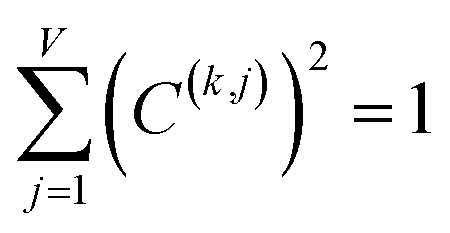
where the subscript *i* represents a datapoint, the superscript (*k*) denotes which principal component (*i.e.*, first, second, third, *etc.*), the superscript *j* denotes which variable, and *V* is the total number of variables.^38^ Because PCA of the covariance matrix is not scale invariant, it should only be used when the various variables are measured on a similar scale (*e.g.*, all variables have the same measurement units).^38,39^

For PCA of the correlation matrix, the eigenvalues sum to the total number of variables.^38^ In this case, the eigenvalues represent how many standardized variables worth of variance are explained by each principal component.^38^ For example, an eigenvalue of 10.3 means that principal component explains as much variance as 10.3 standardized variables. A principal component with an eigenvalue less than one represents less variance than one standardized variable. The goal of PCA is to reduce the number of variables required to explain the data. For PCA of the correlation matrix, the square root of the variance of standardized variables explained by the *k*^th^ principal component (PCk) expands as19
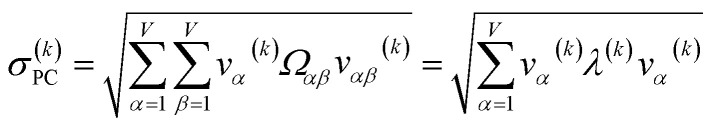
where *v*_*α*_^(*k*)^ is the coefficient for standardized variable *w*^(*α*)^ in the *k*^th^ eigenvector of the correlation matrix, and *λ*^(*k*)^ is corresponding eigenvalue. Because the PC's are normalized20
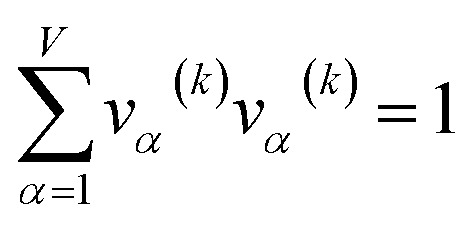
inserting eqn (19) into eqn (20) gives21
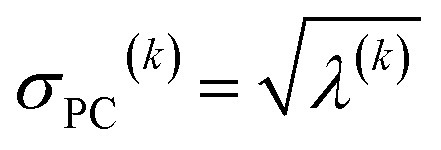


### 2.4 Maximally correlated descriptors

This article focuses on confluence principles for a group of mutually positively correlated descriptors. A set of quantitative descriptors is mutually positively correlated if and only if all elements in the correlation matrix are positive and non-zero

22 *Ω*_*αβ*_ > 0 ∀ *α*, *β*

which is equivalent to all elements in the covariance matrix being positive and non-zero. Although these could represent different experimentally measurable physical properties, the focus in this article is on computed quantitative descriptors that are correlated to many experimentally measurable physical properties but are not themselves uniquely measurable experimentally for most situations. Net atomic charges are a prime example. Centuries of chemical sciences history establish the charges of atoms in materials as a fruitful concept for explaining many chemical phenomena, but various different ways to assign NACs can be conceived.

How does one determine the most suitable definitions for broad use? A definition suitable for broad use should be simultaneously correlated to the various physical properties related to that concept. For example, a NAC definition suitable for broad use should be simultaneously correlated to the experimentally measured chemical properties that are related to the concept of charges of atoms in materials. Such a definition would be a superdelegate that captures the essence of the group of mutually positively correlated descriptors. Because the experimentally measured chemical properties closely related to the concept of charges of atoms in materials must be strongly correlated to some particular NAC definition(s), the superdelegate can be chosen by identifying the group member that maximizes the sum of correlations to group members:23
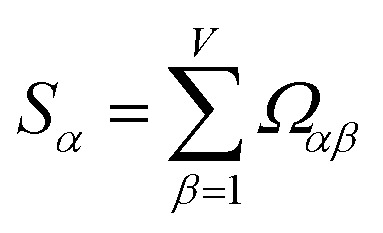
24
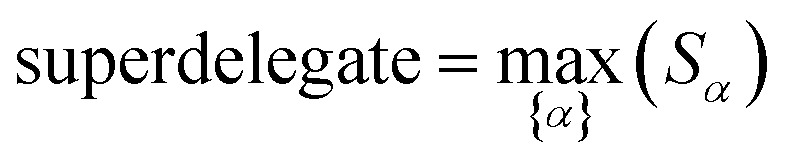


The average standardized variable at datapoint *i* is25
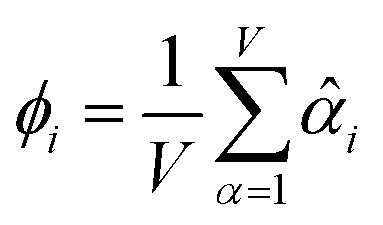
Standardizing this descriptor yields

26 *φ̂*_*i*_ = *ϕ*_*i*_/*σ_ϕ_*

27
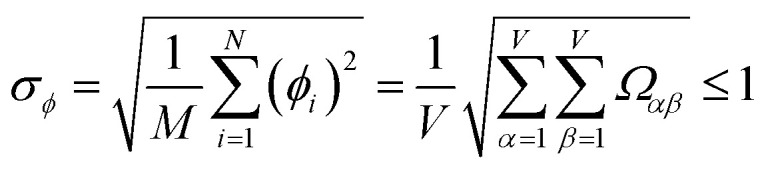


The sum in eqn (23) can be expanded as28
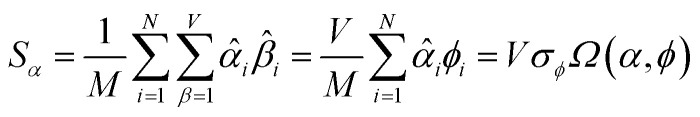
where *Ω*(*α*, *ϕ*) is the correlation between *α* and *ϕ*. Hence, the group member that maximizes the sum of correlations to all group members is the group member that is maximally correlated to the average standardized variable.

As a further performance characteristic, we can ask how correlated this average is to all group members29
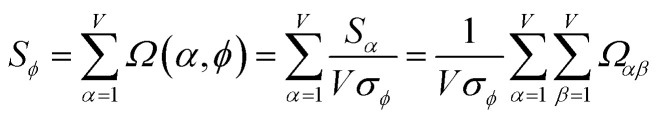
Inserting eqn (27) into eqn (29), this simplifies to30
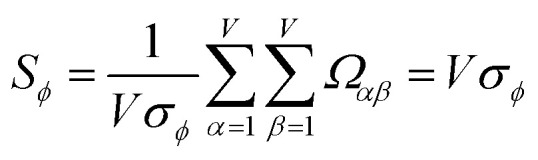


Combining eqn (28) and (30) gives the correlation between standardized variable *α̂* and the average standardized variable *ϕ*:31
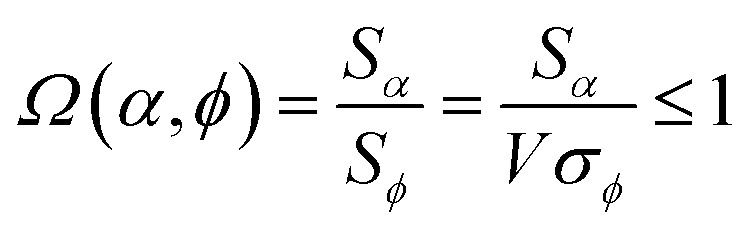
which quantifies the relative ability of standardized variable *α̂* to serve as a delegate for the mutually positively correlated descriptors group.

Section 7.2 below proves that *ϕ* maximizes possible summed correlations to the variables {*α̂*}. That is, *S*_*ϕ*_ ≥ *S*_*τ*_ for any conceivable descriptor *τ* that is a linear combination of the standardized variables.

How is *ϕ* related to the main principal component (MPC) of the correlation matrix? The MPC is the eigenvector with the largest eigenvalue. By definition, a matrix times one of its eigenvectors yields the corresponding eigenvalue (a scalar) times that eigenvector. A common method to find the principal eigenstate is the identity32
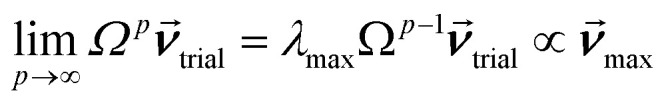
where (***ν⃗***_max_, *λ*_max_) is a principal eigenvector and its eigenvalue. In eqn (32), *p* and *p* − 1 are powers of the matrix. However, eqn (32) only holds if the trial vector is not orthogonal to ***ν⃗***_max_:

33 ·(***ν⃗***_trial_, ***ν⃗***_max_) ≠ 0

Since *φ̂* is maximally correlated to the descriptor group's variables, it is a good initial guess for ***ν⃗***_max_. Substituting *φ̂* for ***ν⃗***_trial_ in eqn (32) yields the first refinement34
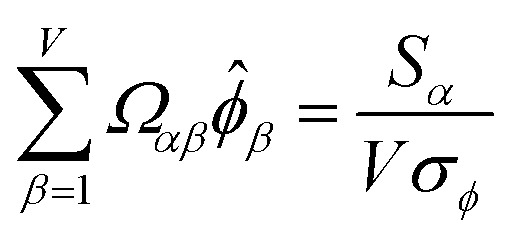
Hence, it follows that the coefficient for standardized variable *α̂* in the MPC for PCA of the correlation matrix is approximately proportional to *S*_*α*_ (*i.e.*, its summed correlation to all variables in the descriptor group). Since the MPC is normalized, this means each variable's coefficient in the MPC is approximately given by35
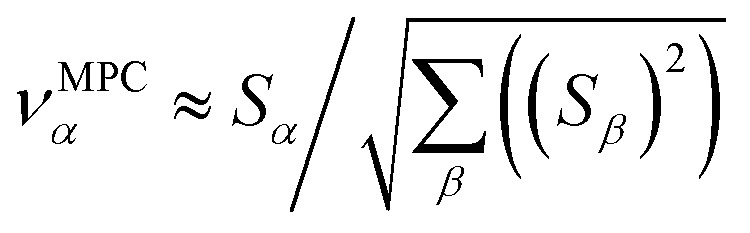


Accordingly, the order of coefficients (largest to smallest) in the MPC of the correlation matrix is approximately the same order as *S*_*α*_ (largest to smallest). Repeated refinement *via*eqn (32) could potentially lead to subtle differences between these two orders, but it is highly unlikely that a bottom 25% variable according to the *S*_*α*_ criterion would become a top 25% variable according to its coefficient in the MPC of the correlation matrix, and *vice versa*.

This analysis clearly reveals a close link between PCA of the correlation matrix, highly correlated descriptors, the average standardized variable *ϕ*, and the superdelegate. Specifically, the superdelegate is the descriptor from the group that has the highest correlation to all group members, and it is likely to have the largest coefficient in the MPC of the correlation matrix. Consequently, this superdelegate will also have relatively high correlation to the MPC of the correlation matrix. Moreover, this superdelegate has high correlation to *ϕ*, and *ϕ* has high correlation to the MPC of the correlation matrix.

## 3. Results

### 3.1 Charge transfer magnitudes and atomic population method classification

Because the average charge transfer magnitude and the correlation matrix are completely independent of each other, both should be considered when assessing the statistical performance of different charge assignment methods. It is possible to have high statistical correlation between two charge assignment methods even though they predict vastly different charge transfer magnitudes. Theoretically, one of these two charge assignment methods could predict reasonable charge transfer magnitudes while the other might severely under-estimate or over-estimate charge transfer magnitudes. This could occur even if the correlation between the two methods is essentially 1.00. Consider two hypothetical methods that assign NACs directly proportional to each other. For example, *A* = 5*B*. The correlation matrix is unchanged if *A* is swapped for *B*. In contrast, the average charge transfer magnitude is directly affected by a scaling factor. In this example, method A has five times the charge transfer magnitude of method B.

The charge transfer magnitude of each NAC method was quantified by its root-mean-squared (rms) deviation from its average value (*i.e.*, *σ* as defined in eqn (1)). Table 1 also lists the average charge, *q*_avg_, for each method across the 29 907 atoms-in-molecules. The small *q*_avg_ differences are due to integration imprecisions. There was a factor of 4.9 between the methods with smallest (*i.e.*, Hirshfeld) and largest (QTAIM) charge transfer magnitudes for molecules. To make the results easier to interpret, the fourth column lists *σ*/*σ*_DDCE6_ as the relative charge transfer magnitude.

**Table 1:** Relative charge transfer magnitudes of 26 NAC methods across ∼2000 molecules and ions. The NAC methods are ordered from smallest to largest charge transfer magnitude. Other characteristics of each NAC method are listed in the remaining columns. The last column includes the following additional comments on convergence properties: (a) non-convex means there is a problem in some materials where the converged solutions are not unique because the optimization landscape is not convex, (b) fails for buried atoms (FFBA) means the method assigns erroneous charges on buried atoms, and (c) frozen core inconsistent (FCI) means the method is defined in such a way that it may give vastly different results if a different number of frozen core electrons is chosen

	*σ*	*q*_avg_	Relative charge transfer magnitude	Basis set limit?	Non-negative density partition?	Approach	Comment
Hirshfeld	0.1284	0.00171	0.413	Yes	Overlapping	Deformation density	
VDD	0.1318	0.00191	0.424	Yes	No	Deformation density	
Mulliken	0.1993	0.00171	0.641	No	No	1PDM projection	
ACP	0.2208	0.00171	0.710	Yes	Overlapping	Dipole intent	FCI
CM5	0.2225	0.00171	0.716	Yes	No	Dipole intent	
ADCH	0.2291	0.00171	0.737	Yes	No	Dipole fit	
EEQ	0.2294	0.00171	0.738	Yes^a^	No	Classical (no QM)	b
i-ACP	0.2994	0.00170	0.963	Yes	Overlapping	Dipole intent	FCI
DDEC6	0.3108	0.00171	1.000	Yes	Overlapping	Confluence	
CHELPG	0.3210	0.00171	1.033	Yes	No	MEP fit	FFBA
IBO	0.3220	0.00171	1.036	Yes	No	Reference orbitals	c
RESP	0.3231	0.00171	1.039	d	No	Constrained MEP fit	d
MK	0.3304	0.00171	1.063	Yes	No	MEP fit	FFBA
Bickelhaupt	0.3345	0.00171	1.076	No	No	1PDM projection	
HLY	0.3465	0.00171	1.115	Yes	No	MEP fit	FFBA
ISA	0.3516	0.00116	1.131	Yes	Overlapping	Spherical averaging	FFBA
Hirshfeld-I	0.3783	0.00171	1.217	Yes	Overlapping	Reference ions	Non-convex
MBIS	0.3808	0.00111	1.225	Yes	Overlapping	Slater functions	Non-convex
MBSBickelhaupt	0.3828	0.00171	1.231	No^e^	No	1PDM projection	
Becke	0.3914	0.00171	1.259	Yes	Overlapping	Reference radii	
Stout–Politzer	0.3937	0.00171	1.267	No	No	1PDM projection	
APT	0.3952	0.00171	1.272	Yes	No	Dipole derivatives fit	
NPA	0.4272	0.00171	1.374	No	No	1PDM projection	
MBSMulliken	0.4333	0.00171	1.394	f	No	1PDM projection	
Ros–Schuit	0.4557	0.00171	1.466	No	No	1PDM projection	
QTAIM	0.6299	0.00171	2.027	Yes	Non-overlapping	Viral compartments	g

a No basis set or quantum chemistry calculation is required to compute EEQ NACs.

b Many different charge electronegativity equilibration schemes have been proposed. Many of these are not robust, because they sometimes produce extremely high NAC magnitudes.

c The IBO method currently requires the first-order density matrix to be idempotent.

d Whether or not the RESP NACs have a complete basis set limit depends on the type of fitting constraints used. If and only if the fitting constraints have no basis set dependence or have a complete basis set limit, then the corresponding RESP NACs will have a complete basis set limit. Whether the RESP NACs are robust depends on how the constraints are constructed.

e Not rotationally invariant.

f Methods that project populations from a quantum chemistry calculation basis set (aka ‘source basis set’) onto a small basis set (aka ‘target basis set’) have a basis set limit with respect to improving the source basis set towards completeness, but their results depend on the small target basis set onto which the populations are projected.

g QTAIM partitions are robust only when they have been sufficiently smoothed so that noise does not create spurious virial compartments.

The fifth column of Table 1 indicates whether the NACs have a mathematical limit as the basis set is improved towards completeness. Individual atom-in-material descriptors (*e.g.*, net atomic charges, atomic spin moments (ASMs), bond orders, spdfg populations, polarizabilities, *etc.*) only have clear chemical and physical meaning when they converge to well-defined values as the basis set is improved (*i.e.*, they have complete basis set limits). Therefore, population analysis methods lacking a complete basis set limit are not useful for computing these properties. Regardless of whether or not an atomic population analysis method has a complete basis set limit, it can still act as a useful basis representation to expand quantum mechanical operators. For example, the electron–electron Coulomb electrostatic energy of a material can be expressed exactly as a polyatomic multipole expansion plus charge overlap terms. This Coulomb energy can be expanded exactly using any population analysis method that reproduces the material's electron distribution, irrespective of whether that population analysis method has a complete basis set limit. However, when the population analysis method lacks a complete basis set limit it is only the computed coulombic energy and not the individual populations that carry any physical meaning. Consequently, individual values of atom-in-material descriptors reported in scientific publications should be computed using methods having a complete basis set limit.

A complete basis set limit is a necessary but not a sufficient condition for computing highly valuable atom-in-material descriptors. Several other criteria are also required: (a) the atom-in-material descriptor values should be highly correlated to many experimentally measured properties, (b) the population analysis method should yield a correct atom-in-material descriptor value for carefully chosen benchmark systems having well-known and unambiguous atom-in-material properties, and (c) the population analysis method should yield atom-in-material descriptor values that are chemically consistent amongst themselves (*e.g.*, the NAC value should be chemically consistent with the ASM value^15^). These and related criteria are explained more fully in Section 5.

Every quantum chemistry calculation in which the electron density is properly computed from a wavefunction yields a non-negative total electron density36
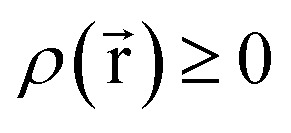
(**Caution**: Quantum chemistry algorithms that merely estimate the electron density using response theory may yield 
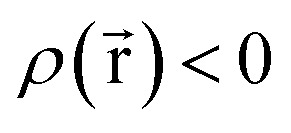
 for some position 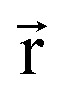; this does not correspond to the proper electron density of any wavefunction.) The sixth column of Table 1 indicates whether each method partitions the total electron density37
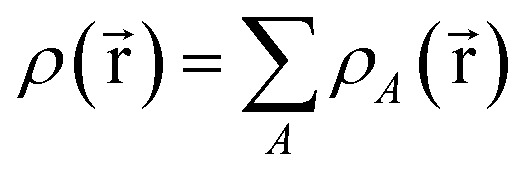
into non-negative atom-in-material electron densities38
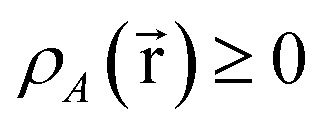
having a complete basis set limit. If yes, then partitioning into overlapping versus non-overlapping 
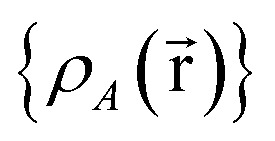
 is indicated. “No” means either that the electron density is not partitioned, that some partitions can have negative density values at some spatial positions, or that the method lacks a complete basis set limit. The electron density partitions in eqn (37) usually correspond to atoms, but the QTAIM method can have some non-nuclear attractors (*i.e.*, one or more electron density partitions that are not atoms).^46,47^ Such non-nuclear attractors are a modeling advantage for electrides but can be a modeling disadvantage for other materials.^15,48^

The seventh column in Table 1 briefly summarizes the charge assignment strategy. The Hirshfeld and VDD methods partition the molecule's deformation density into overlapping and non-overlapping partitions, respectively. Methods marked “1PDM projection” project components of the one-particle density matrix (1PDM). Methods marked “dipole intent” were developed to approximately reproduce the molecular dipole moments of reference compounds. “Dipole fit” indicates the NACs are optimized to reproduce each molecule's quantum-mechanically computed dipole moment. The EEQ method requires no quantum chemistry calculation. “MEP fit” indicates the NACs minimize some error measure between the quantum-mechanical molecular electrostatic potential (MEP) and the electrostatic potential of the NAC model; these methods may differ by the grid points and integration weights used to construct the error measure. The DDEC6 method optimizes NACs to simultaneously give small errors across both electrostatic and chemical properties. The APT method optimizes the NACs to reproduce changes in the molecular dipole moment as the atoms vibrate, assuming each NAC is constant as the molecule vibrates.^10^ Entries marked “reference orbitals”, “reference ions”, “reference radii”, “spherical averaging”, and “Slater functions” indicate a key feature of the charge assignment scheme. The QTAIM method assigns non-overlapping virial compartments.^32,49–51^

Cho *et al.* misclassified the DDEC6 method as an “iterative Hirshfeld variant” (page 694 of ref. 1), which it is not. The Hirshfeld and VDD approaches are based on deformation density partitioning using overlapping and non-overlapping compartments, respectively.^21,36^ As shown in Table 1, deformation density approaches yield the lowest average charge transfer magnitudes of all charge assignment methods. The iterative Hirshfeld (aka Hirshfeld-I) method was developed by Bultinck *et al.* and sets the atomic weighting function equal to a quantum-mechanically computed reference ion density, where the reference ion's charge is self-consistently updated to match the assigned AIM charge.^25^ The earliest DDEC methods used a combination of spherical averaging and charge-compensated reference ions for which the reference ion charges were self-consistently updated to match the assigned AIM charges.^52^ Unfortunately, the Hirshfeld-I and early DDEC methods suffer the runaway charges problem in which vastly different NACs are sometimes assigned to symmetry equivalent atoms in materials.^15,53^ The DDEC6 method uses a fixed sequence of seven charge partitioning steps to solve the runaway charges problem.^15^

DDEC6 is the sixth generation improvement of the Density-Derived Electrostatic and Chemical (DDEC) methods.^15,54–56^ DDEC6 uses: (a) tail constraints on the atomic weighting functions to prevent them from becoming too diffuse or contracted for buried atom tails, (b) reference ion charges that approximate the number of electrons in the volume dominated by each atom, (c) reference ion smoothing and conditioning to allow the reference ions to expand or contract according to the material's local environment, (d) a weighted spherical average to more accurately reproduce the electrostatic potential surrounding the material, and (e) a fixed sequence of seven charge partitioning steps to avoid the runaway charges problem.^15,53^

The last column in Table 1 includes comments on specific convergence issues. Methods that can converge to vastly different solutions depending on the initial guess do not have a convex optimization functional for some materials; the Hirshfeld-I and MBIS methods are such examples.^15,27^ Methods with a convex optimization landscape that is nearly flat for buried atoms can assign buried atom charges that are not chemically meaningful; the CHELPG, HLY, ISA, and MK electrostatic potential fitting methods are such examples.^23,33,52^ Many different charge electronegativity equilibration schemes have been proposed.^16–18,20,57–61^ Many charge electronegativity equilibration schemes sometimes produce extremely high NAC magnitudes.^58,61,62^ The ACP and i-ACP NACs are sensitive to the choice of valence electrons for each chemical element; for example, vastly different results might be obtained depending on whether Cs element is considered to have one (*i.e.*, 6s^1^) or nine (*i.e.*, 5s^2^5p^6^6s^1^) valence electrons. This unfortunate dependency arises, because the ACP and i-ACP methods are defined to fit the entire valence electron population of an atom-in-material using only one Slater exponential decay function.^8,24^ [CsO_4_]^+^ has strong polar-covalent bonding between the Cs and O atoms not purely ionic bonding.^63^ In [CsO_4_]^+^, the 5s and 5p ‘semi-core’ electrons are key participants in the polar-covalent bonding, thus acting as valence electrons along with higher subshells.^63^

Examining Table 1, the deformation density methods (*i.e.*, Hirshfeld and VDD) had the smallest charge transfer magnitudes, while partitioning based on Virial compartments (*i.e.* QTAIM) had the largest. Methods designed to approximately (*i.e.*, ACP, CM5, i-ACP) or exactly (*i.e.*, ADCH) reproduce the molecular dipole moment had larger average charge transfer magnitudes than the deformation density group but smaller than the MEP fitting group (CHELPG, RESP, MK, HLY). The DDEC6, IBO, Bickelhaupt, and ISA methods had average charge transfer magnitudes similar to the MEP fitting group. Many methods (*e.g.*, Hirshfeld-I, MBIS, Becke, APT, *etc.*) had average charge transfer magnitudes larger than the MEP fitting group.

As an illustrative example, Table 2 summarizes selected calculations for the water molecule. Water was chosen for two reasons. First, it participates in many biological, environmental, geological, and chemical processes. Second, its three-atom bent geometry permits NACs to be directly derived from its calculated molecular dipole moment. This corresponds to the ADCH oxygen NAC of −0.693. Larger molecules containing more than two distinct atom types do not have uniquely determined NACs derived only from the molecule's dipole moment, because multiple NAC values could reproduce the same molecular dipole moment. The CM5 oxygen NAC of −0.642 was slightly smaller in magnitude than the ADCH value. All four MEP fitting methods (CHELPG, HLY, MK, and RESP) yielded practically identical oxygen NAC of −0.715 to −0.704. Moreover, the oxygen NAC that minimized the RMSE over the 788833 grid points for data listed in Table 2 was also within this same range. The DDEC6 (−0.802) and Hirshfeld-I (−0.900) oxygen NACs were somewhat larger in magnitude than the MEP fitting group. As expected, the deformation density (*i.e.*, Hirshfeld and VDD) NACs were too small in magnitude to approximate the molecular dipole moment or MEP. Also as expected, the QTAIM NACs were too large in magnitude to approximate the molecular dipole moment or MEP. When atomic dipoles are included, the molecular dipole moment is reproduced exactly.

**Table 2:** Relative root mean squared errors (RRMSE) in electrostatic potential of the water molecule for 20 charge assignment methods having a complete basis set limit. Errors in the predicted molecular dipole moment magnitude are also listed. Methods listed from smallest to largest NAC magnitude on oxygen. For some of the non-negative AIM density partitioning methods, the errors including atomic dipoles are listed in parentheses

Method	Oxygen NAC	RRMSE (%)	Δ*μ* (%)
VDD	−0.286	61%	−59%
Hirshfeld	−0.306	58% (11%)	−56% (0%)
EQeq	−0.368	49%	−47%
APT	−0.513	30%	−26%
ACP	−0.522	29%	−25%
CM5	−0.642	16%	−7%
Becke	−0.645	16% (22%)	−7% (0%)
MBSMulliken	−0.663	15%	−4%
ADCH	−0.693	14%	0%
RESP	−0.704	14%	2%
MK	−0.705	14%	2%
CHELPG	−0.710	14%	2%
HLY	−0.715	14%	3%
i-ACP	−0.720	14%	4%
IBO	−0.734	14%	6%
DDEC6	−0.802	19% (8%)	16% (0%)
ISA	−0.841	23% (7%)	21% (0%)
MBIS	−0.876	27% (6%)	26% (0%)
Hirshfeld-I	−0.900	30% (4%)	30% (0%)
QTAIM	−1.212	72% (10%)	75% (0%)

The data in Table 2 were computed as follows. The optimized molecular geometry, electron density distribution, and reference electrostatic potential were computed using Gaussian 16 (ref. 64) software. The dipole moment magnitude of the computed PBE0/def2TZVPP optimized geometry and electron density was 0.765 au, which was used as the reference dipole moment. Using an in-house program, the RRMSE was computed over a uniform grid of 788833 points between 1.4–2.0 times the van der Waals radii. (vdW radii values for *H* = 2.73 and *O* = 3.31 bohr.) The RRMSE is a percentage of the root mean squared error (RMSE) for a zero charge model (RMSE = 8.72 kcal mol^−1^). The ADCH, Becke, CHELPG, MK, QTAIM, RESP, and VDD charges were computed with Multiwfn^65^ version 3.6. The CM5, DDEC6, and Hirshfeld charges were computed using the Chargemol^55^ program. The Hirshfeld-I, ISA, and MBIS charges were computed using a modified in-house Chargemol version. The APT, HLY (keyword = HLYGat), and MBSMulliken charges were computed in Gaussian 16. The EQeq charges were computed using Racek *et al.*'s online calculator^66^ using Wilmer *et al.*'s^20^ method. The IBO charges were computed using Knizia's IBOView version 20150427.^22,67^ The ACP and i-ACP charges were computed using the ACP^8,68^ and i-ACP^24,69^ programs. Although the ACP and i-ACP methods could potentially be used to compute atomic dipoles, these were not available in the software versions used.

### 3.2 Identifying highly correlated descriptors

**Fig. 2:**
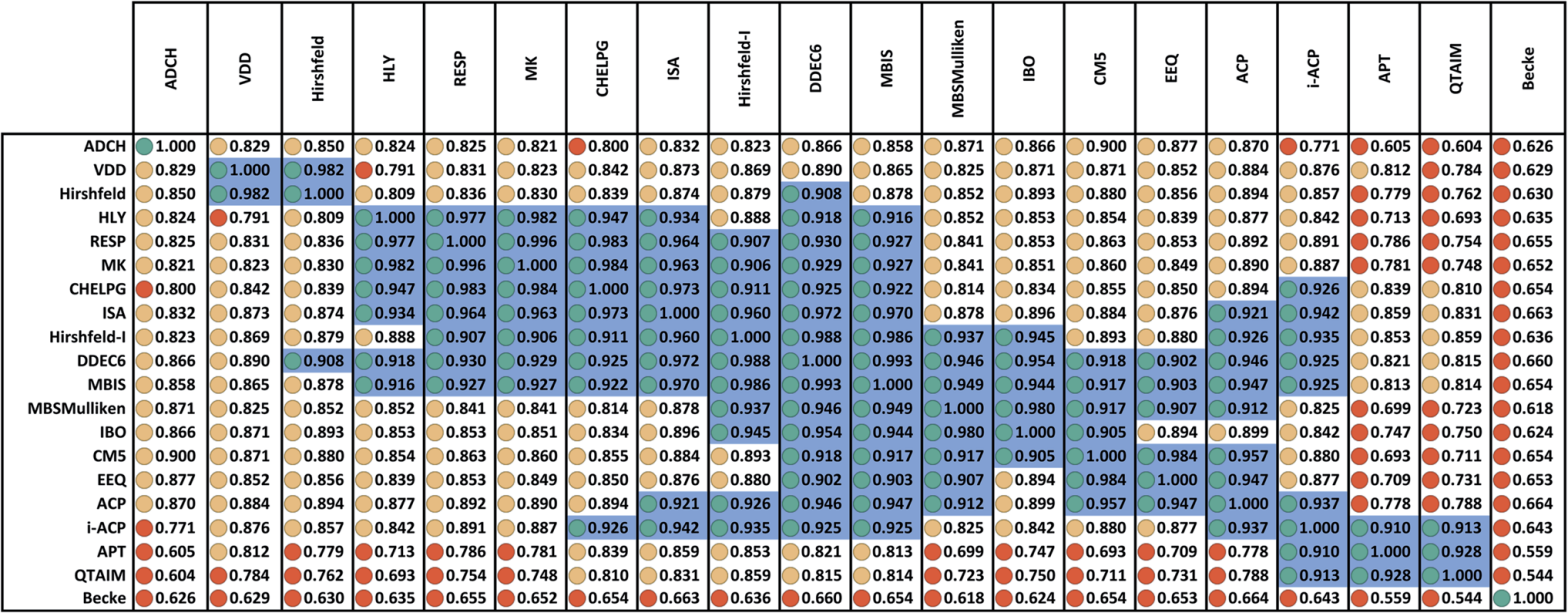
Correlation matrix between 20 methods having a complete basis set limit for assigning net atomic charges in molecules. Stoplight colors indicate the covariance values: green ≥ 0.9, 0.8 ≤ yellow < 0.9, red < 0.8. Blue shading marks blocks of values ≥ 0.9. There are three primary groups: (a) a main group that covers a large number of methods, (b) the i-ACP, APT, and QTAIM group, and (c) the VDD and Hirshfeld group. The DDEC6 method is strongly correlated to all members of group (a) plus the i-ACP method in group (b) and the Hirshfeld method in group (c). No other charge assignment method besides DDEC6 is strongly correlated to some members of all three groups. The ADCH and Becke methods are not strongly correlated to any charge assignment methods besides self. The ADCH-CHELPG entry is red rather than yellow, because its value is 0.7996 which is below the 0.8 cutoff. The ADCH-CM5 entry is yellow rather than green, because its value is 0.8999 which is below the 0.9 cutoff.

Fig. 2 displays the correlation matrix between all 20 methods having a complete basis set limit. As explained in Section 2.2 above, this also equals the covariance matrix of the standardized variables. Fig. 2 is related to Table 2 of Cho *et al.* that displayed the squared correlation matrix for 18 of the 26 methods.^1^ (The source data for both was similar, except Fig. 2 incorporates the minor corrections noted in Section 2.1 above.) Analogous to Cho *et al.*'s approach, Fig. 2 arranges highly correlated methods close to each other. Blue shading marks blocks of methods having correlation ≥ 0.9.

The Becke method had extremely low correlation (<0.7) to the 19 other methods. In fact, Becke introduced an integration algorithm (ref. 11) not a method to compute NACs; the Becke NACs were introduced by later authors who misapplied Becke's integration algorithm. This is why Becke NACs are poorly correlated. The DDEC6 method connects 15 of the 20 methods. Only the ADCH, APT, Becke, QTAIM, and VDD methods have correlation < 0.9 to the DDEC6 method. Excluding Hirshfeld, the remaining 14 methods connected to DDEC6 form the main block. ADCH is almost connected to the main block through the ADCH-CM5 correlation = 0.8999, but it has no correlation ≥ 0.9 to any method except self. A small side block containing the deformation density methods (Hirshfeld and VDD) is connected to the main block only through the Hirshfeld-DDEC6 correlation = 0.908. Another small side block containing i-ACP (also part of the main block), APT, and QTAIM is connected to the main block through i-ACP. Within the main block, DDEC6 is the most connected (15 correlations ≥ 0.9) and IBO and EEQ are the least connected (each having 6 correlations ≥ 0.9).

Several atomic population analysis methods optimize similarity between atom-in-material electron distributions and those of quantum-mechanically computed reference atoms. These methods require a library of quantum-mechanically computed reference atoms. Among the 26 atomic population analysis methods considered here, these include DDEC6, Hirshfeld, Hirshfeld-I, and IBO. Only the neutral uncharged ground-state reference atoms are required for the Hirshfeld and IBO methods, while ground-state reference ions in various charge states are required for the DDEC6 and Hirshfeld-I methods. The IBO method uses an ingenious projection to represent the molecular orbitals in terms of polarized atom-in-material orbitals.^22^ Currently, the IBO method is limited to idempotent density matrices.^22^ The DDEC methods use charge-compensated reference ions and reference ion conditioning to polarize reference ions by their material environment.^15,52,53^ The similar average charge transfer magnitudes (see Table 1) of DDEC6 and IBO NACs is notable. As shown in Fig. 2, the correlation between DDEC6 and IBO NACs is 0.954. Although the Hirshfeld and IBO methods are both based on neutral uncharged reference atoms, their average charge transfer magnitudes differ by a factor of 2.5. The average charge transfer magnitude of the Hirshfeld-I method is about 1.2 times that of the DDEC6 method. Correlation between the DDEC6 and Hirshfeld-I methods is high at 0.988. In spite of the similar names, the Hirshfeld NACs have slightly less correlation to the Hirshfeld-I NACs (0.879) than to both the IBO (0.893) and DDEC6 (0.908) NACs.

Table 3 summarizes PCA of the correlation matrix. All methods had positive coefficients in the MPC. The 14 methods of the main block had the largest MPC coefficients. Comparing columns 2 and 6 of Table 3 shows the approximation of eqn (35) is almost exact. The eigenvalue shows the MPC accounts for 17.158 variables' (85.8%) worth of correlation. This clearly reflects the size of the main block (14 methods) plus some contributions from small side blocks weakly connected to the main block. The other principal components (*i.e.*, PC2, PC3, PC4, *etc.*) account for less than one variable's worth of correlation apiece.

**Table 3:** The first four eigenvalues and principal components coefficients for correlation PCA of 20 charge assignment methods having a complete basis set limit. The methods are listed in order from largest to smallest contribution to the MPC. The last column is listed for comparison to the MPC coefficient of column 2

	PC1 (MPC)	PC2	PC3	PC4	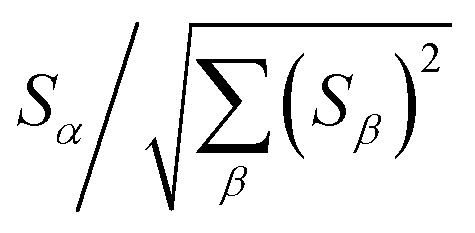
% correlation explained	85.8%	4.1%	2.8%	2.6%	—
Eigenvalue→	17.158	0.816	0.562	0.524	—
DDEC6	0.238	0.020	−0.035	−0.075	0.238
MBIS	0.237	0.020	−0.015	−0.098	0.237
ISA	0.236	−0.094	0.142	−0.102	0.236
Hirshfeld-I	0.235	−0.076	−0.082	−0.078	0.235
ACP	0.233	0.083	−0.097	0.023	0.233
CHELPG	0.230	−0.126	0.301	−0.134	0.230
i-ACP	0.230	−0.249	−0.043	0.046	0.230
RESP	0.230	−0.011	0.340	−0.185	0.229
MK	0.229	−0.007	0.354	−0.200	0.229
IBO	0.228	0.156	−0.220	−0.043	0.227
CM5	0.227	0.247	−0.145	0.030	0.227
EEQ	0.225	0.207	−0.146	0.050	0.225
MBSMulliken	0.225	0.226	−0.203	−0.072	0.225
HLY	0.224	0.094	0.366	−0.246	0.224
Hirshfeld	0.223	0.045	−0.259	0.123	0.223
VDD	0.222	−0.026	−0.263	0.158	0.222
ADCH	0.213	0.376	−0.082	0.004	0.213
APT	0.204	−0.537	−0.087	0.096	0.205
QTAIM	0.203	−0.514	−0.189	0.106	0.203
Becke	0.169	0.126	0.420	0.861	0.171

Confluence, which will be more thoroughly explained in Section 4 below, occurs when a quantitative descriptor yields high correlations across a broad group of related descriptors and physical properties. Here, there are three key indicators that confluence occurs among NAC descriptors. The first, and perhaps most important, is the MPC accounts for the vast majority (*i.e.*, 85.8%) of correlation within this NAC descriptor group. The second is that each of the remaining principal components is extremely weak, accounting for less than one variable's worth of correlation apiece. The third is that at least one individual NAC descriptor (*e.g.*, DDEC6) is highly correlated to a large percentage (*e.g.*, 15/20 = 75%) of NAC descriptors. Of course, the correlation matrix's pronounced main block illustrated within Fig. 2 is a consequence of these three factors.

Since confluence is present within this group of NAC descriptors, it is useful to ask: “Which individual member of the group best represents the group as a whole?” Different criteria can be conceived to determine this: (1a) the member having the largest coefficient in the correlation MPC, (1b) the member having the highest correlation to the correlation MPC, (2a) the member having the highest summed correlation to all group members (*i.e.*, largest *S*_*α*_), (2b) the member having the highest correlation to the average standardized variable *ϕ* (*i.e.*, largest *Ω*_(*αϕ*)_), or (3) the member having strong correlations to the largest number of other group members. Because criteria (1a) and (1b) are proportional to each other (see confluence principle # 2 of Section 4), they always give identical rankings. Because criteria (2a) and (2b) are proportional to each other (see eqn (28)), they always give identical rankings. By eqn (35), rankings according to criteria (1a) and (2a) will be similar but not necessarily identical. Clearly, a member that has strong correlations to a large number of other group members must also have a relatively high *S*_*α*_; therefore, criteria (2a) and (3) often give somewhat similar results. Consequently, in practice the results are often similar irrespective of which criterion is chosen.

Table 3 ranks NAC methods according to criterion (1a). Table 4 ranks NAC methods according to criterion (1b), (2a), (2b), and (3). Rankings for criterion (3) were performed separately using two different thresholds: the numbers of NAC methods having correlation ≥0.8 and ≥ 0.9 to each method. The top (DDEC6), the bottom (Becke), the 2^nd^ (MBIS), the 8^th^ (RESP), the 9^th^ (MK), the 11^th^ (CM5), and the 15^th^ (Hirshfeld) ranked methods had consistent rankings across all ranking criteria. Across the different ranking criteria, small variations in the placements of other methods were observed.

**Table 4:** Rank of each charge assignment method according to its amount of correlation to other charge assignment methods. The *S*_*α*_ and *Ω*(*α*, *ϕ*) ranking criteria always give the same order of methods. This table includes 20 charge assignment methods with a complete basis set limit

Rank	Method	*S*_*α*_	*Ω* (*α*, *ϕ*)	Method	*Ω* (*α*, MPC)	Method	Number (*Ω*_*αβ*_ > 0.8)	Method	Number (*Ω*_*αβ*_ > 0.9)
1	DDEC6	18.204	0.985	DDEC6	0.986	DDEC6	19	DDEC6	15
2	MBIS	18.109	0.980	MBIS	0.981	MBIS	19	MBIS	14
3	ISA	18.064	0.977	ISA	0.978	ISA	19	Hirshfeld-I	11
4	Hirshfeld-I	17.981	0.973	Hirshfeld-I	0.974	Hirshfeld-I	19	ISA	10
5	ACP	17.823	0.964	ACP	0.965	CHELPG	18	ACP	9
6	i-ACP	17.603	0.953	CHELPG	0.953	i-ACP	18	CHELPG	9
7	CHELPG	17.600	0.952	i-ACP	0.952	ACP	17	i-ACP	9
8	RESP	17.564	0.950	RESP	0.951	RESP	17	RESP	8
9	MK	17.520	0.948	MK	0.949	MK	17	MK	8
10	IBO	17.400	0.942	IBO	0.942	IBO	17	MBSMulliken	8
11	CM5	17.396	0.941	CM5	0.942	CM5	17	CM5	7
12	EEQ	17.237	0.933	EEQ	0.933	EEQ	17	HLY	7
13	MBSMulliken	17.188	0.930	MBSMulliken	0.931	MBSMulliken	17	IBO	6
14	HLY	17.143	0.928	HLY	0.929	VDD	17	EEQ	6
15	Hirshfeld	17.088	0.925	Hirshfeld	0.924	Hirshfeld	17	Hirshfeld	3
16	VDD	16.996	0.920	VDD	0.919	HLY	16	APT	3
17	ADCH	16.318	0.883	ADCH	0.883	ADCH	15	QTAIM	3
18	APT	15.683	0.849	APT	0.847	APT	9	VDD	2
19	QTAIM	15.562	0.842	QTAIM	0.840	QTAIM	8	ADCH	1
20	Becke	13.052	0.706	Becke	0.699	Becke	1	Becke	1

Cho *et al.* previously reported MBSBickelhaupt and Hirshfeld-I as having largest correlation to the unstandardized covariance MPC among 16 NAC methods.^1^ Because the unstandardized covariance matrix is sensitive to multiplying a variable by a scale factor, PCA of the unstandardized covariance matrix tends to favor contributions from variables having larger *σ*, when compared to PCA of the correlation matrix. Because our goals in this paper are to examine the charge transfer magnitudes and correlation properties, we refer readers to Cho *et al.*'s work^1^ for a detailed discussion of PCA of the unstandardized covariance matrix.

Returning to a discussion of Table 4, it is instructive to ask how well DDEC6 performs compared to the correlation MPC and compared to the average standardized variable *ϕ*. Among all conceivable descriptors, *S*_max_ = *S*_*ϕ*_ = 18.4806 is the highest possible sum of correlations to the 20 NAC methods. The sum of correlations between the MPC and the NAC methods is *S*_MPC_ = 18.4795 and almost as high as *S*_*ϕ*_. The *S*_DDEC6_ = 18.204 is 0.985 times *S*_*ϕ*_, which also equals the correlation between DDEC6 NAC and *ϕ*. Correlation between DDEC6 and correlation MPC is almost the same at 0.986. Hence, the DDEC6 NAC captures much of the same information that is captured by *ϕ* and the correlation MPC.

Examining other high performing methods, the top four ranked methods have *S*_DDEC6_ − *S*_*α*_ < *S*_max_ − *S*_DDEC6_, while this inequality does not hold for methods ranked fifth and beyond. Hence, the top four ranked methods (*i.e.*, DDEC6, MBIS, ISA, and Hirshfeld-I) have relatively small differences between their *S*_*α*_ values. Consequently, the average charge transfer magnitudes should also be considered when selecting among these four methods. Among these four methods, the average charge transfer magnitudes from Table 1 are DDEC6 (1.000) < ISA (1.131) < Hirshfeld-I (1.217) ≈ MBIS (1.225). Average charge transfer magnitudes of the Hirshfeld-I and MBIS methods are arguably a bit too high, especially if the goal is to use a NAC model to approximately reproduce the MEP surrounding the molecule.

### 3.3 Sensitivity of ranking to the choice of included methods

A key question is “How robust are the rankings of the top-ranked methods to changes in which other methods are included in the dataset?” For example, what happens if the dataset is spammed with trivial variations of one charge assignment method? For example, electrostatic potential fitting methods such as CHELPG, HLY, and MK differ only in the choice and weighting of grid points on which the root mean squared error (RMSE) of the electrostatic potential is computed and minimized. With slightly different choices in the grid points and their weightings, one could easily produce a thousand slightly different variations of electrostatic potential fitting methods. If these are included in the dataset would they force one of the electrostatic potential fitting methods into the top-ranked position? Somewhat surprisingly, the answer is no. Spamming the database with trivial variations of one method is not sufficient to elevate that method into the top-ranked position if the method being spammed is highly correlated (*i.e.*, *Ω*_*αβ*_ > 0.9) to the top-ranked method. For some of the ranking criteria, the top-ranked charge assignment method does not change under such a scenario. Examining Fig. 2 and Table 4, the DDEC6 method is highly correlated (*i.e.*, *Ω*_*αβ*_ > 0.9) to 15 charge assignment methods, including the CHELPG method which is highly correlated to 9 charge assignment methods. If 1000 new electrostatic potential fitting methods that are trivial variations compared to CHELPG are added to the dataset, this increases the number of methods highly correlated to both DDEC6 and CHELPG by exactly 1000. The new numbers of highly correlated methods (1015 to DDEC6 and 1009 to CHELPG) do not change the relative order of these two methods at all. This new dataset yields rankings identical to the original dataset for each of the top 16 methods according to the *Ω*_*αβ*_ > 0.8 ranking criterion and for each of the top 4 methods according to the *Ω*_*αβ*_ > 0.9 ranking criterion.

Moreover, such spamming can be easily detected by a ranking abnormality. In the above example, *S*_*α*_ for CHELPG would increase from 17.600 in the original dataset to 1017.600 in the modified dataset, while *S*_*α*_ for DDEC6 would increase from 18.204 to (18.204 + 1000 × 0.9252) = 943.4. The better ranking of DDEC6 than CHELPG for number of methods with *Ω*_*αβ*_ > 0.9 and *Ω*_*αβ*_ > 0.8 but worse ranking of DDEC6 compared to CHELPG for *S*_*α*_ in the modified dataset is a clear indication the modified dataset contains a cluster of methods highly similar to CHELPG which are not as confluent as DDEC6 across the entire database. In other words, this ranking abnormality (*i.e.* different top-ranked method for *S*_*α*_ criterion compared to *Ω*_*αβ*_ > 0.9 criterion) makes the spamming obvious and easy to detect.

What happens if the method being spammed is low-ranked in the original dataset? For example, if 1000 trivial variations of the Becke method were added to the dataset? Since the Becke method has low correlations to all of the other charge assignment methods in the original dataset, this spamming would force all of these trivial variations of the Becke method into the top-ranked positions of the modified dataset for any of the ranking criteria used in Table 4. However, it would be easy to detect this sham confluence. When genuine confluence occurs, the confluent method exhibits confluence not only across various computed descriptors but also across the physical properties those computational descriptors are intended to describe. Although the Becke method performed well for the water molecule (see Table 2), it gave the wrong sign and magnitude of NAC for Eu in [Eu@C_60_]^+^ (see Table 7). Specifically, the Becke NAC of −4.427 for the Eu atom in [Eu@C_60_]^+^ is chemically wrong. Also, Table 1 shows the average charge transfer magnitude of the Becke method is relatively high compared to methods optimized to reproduce the MEP.

Another important question is whether the rankings would remain similar if new methods are added to the dataset that are not trivial variations of the already included methods. Moreover, will the rankings be adversely affected if the quality of these newly added methods is dubious? To address this question, the dataset is re-analyzed by adding the six charge assignment methods that do not have a complete basis set limit. Comparing the new rankings listed in Table 5 to the original rankings in Table 4, the DDEC6 and MBIS methods remain in the first and second spots, respectively, for all of the metrics. The MBSBickelhaupt method (which is one of the newly added methods) is now in the third spot according to all the metrics. Hirshfeld-I now places fourth according to all the metrics, while it originally placed fourth according to all the metrics except one for which it originally placed third. This analysis shows the relative rankings of the methods are only weakly affected by adding a modest number of new methods, even if those new methods are of dubious quality.

**Table 5:** Rank of each charge assignment method according to its amount of correlation to other charge assignment methods. The *S*_*α*_ and *Ω*(*α*, *ϕ*) ranking criteria always give the same order of methods. This table includes all 26 charge assignment methods

Rank	Method	*S*_*α*_	*Ω*(*α*, *ϕ*)	Method	*Ω*(*α*, MPC)	Method	Number (*Ω*_*αβ*_ > 0.8)	Method	Number (*Ω*_*αβ*_ > 0.9)
1	DDEC6	23.575	0.986	DDEC6	0.987	DDEC6	24	DDEC6	20
2	MBIS	23.481	0.982	MBIS	0.983	MBIS	24	MBIS	19
3	MBSBickelhaupt	23.468	0.981	MBSBickelhaupt	0.981	MBSBickelhaupt	24	MBSBickelhaupt	16
4	Hirshfeld-I	23.251	0.972	Hirshfeld-I	0.973	Hirshfeld-I	24	Hirshfeld-I	14
5	ISA	23.195	0.970	ISA	0.970	ISA	24	ACP	14
6	ACP	23.138	0.967	ACP	0.967	Bickelhaupt	24	Bickelhaupt	14
7	Bickelhaupt	23.093	0.965	Bickelhaupt	0.966	i-ACP	23	ISA	13
8	NPA	22.884	0.957	NPA	0.958	ACP	22	MBSMulliken	13
9	IBO	22.801	0.953	IBO	0.954	NPA	22	Mulliken	13
10	CM5	22.693	0.949	CM5	0.949	IBO	22	NPA	12
11	MBSMulliken	22.663	0.947	MBSMulliken	0.948	CM5	22	IBO	11
12	Mulliken	22.653	0.947	Mulliken	0.947	MBSMulliken	22	CM5	11
13	EEQ	22.540	0.942	EEQ	0.942	Mulliken	22	i-ACP	10
14	i-ACP	22.530	0.942	i-ACP	0.942	EEQ	22	CHELPG	10
15	RESP	22.467	0.939	RESP	0.940	RESP	22	Stout–Politzer	10
16	CHELPG	22.429	0.938	CHELPG	0.938	CHELPG	22	EEQ	8
17	MK	22.414	0.937	MK	0.938	MK	22	RESP	8
18	Stout–Politzer	22.162	0.927	Stout–Politzer	0.927	Hirshfeld	22	MK	8
19	Hirshfeld	22.074	0.923	Hirshfeld	0.923	HLY	21	HLY	7
20	HLY	22.021	0.921	HLY	0.922	VDD	21	Hirshfeld	4
21	VDD	21.897	0.915	VDD	0.915	Stout–Politzer	20	APT	3
22	ADCH	21.283	0.890	ADCH	0.890	ADCH	20	QTAIM	3
23	APT	19.970	0.835	APT	0.834	APT	11	VDD	2
24	QTAIM	19.855	0.830	QTAIM	0.830	QTAIM	10	ADCH	1
25	Ros–Schuit	16.867	0.705	Ros–Schuit	0.701	Ros–Schuit	1	Ros–Schuit	1
26	Becke	16.718	0.699	Becke	0.693	Becke	1	Becke	1

Another useful question is whether the data for one particular method has the potential ability to dramatically alter the rankings. A way to frame this question is to ask how the rankings could potentially change if one of the charge assignment methods in the original dataset is swapped for a new charge assignment method having any conceivable properties. Examining Table 4, the number of (*Ω*_*αβ*_ > 0.9) ranking criterion is the most robust to this kind of method swap. For charge assignment method A, swapping one of the other charge assignment methods (B) for an arbitrary new one (B′) could affect the number methods having (*Ω*_*αβ*_ > 0.9) to method A by: (i) +1 if method B′ is highly correlated to method A while method B is not, (ii) by −1 if method B is highly correlated to method A while method B′ is not, and (iii) otherwise this number will be unchanged by the swap. Examining Table 4, a change in ±1 in the number of (*Ω*_*αβ*_ > 0.9) for each method would leave DDEC6 and MBIS in either the first or second spots. Hence, any conceivable change to a single charge assignment method only has a small potential impact on the (*Ω*_*αβ*_ > 0.9) ranking criterion.

Finally, consider the grouping of methods into families of related methods. The electrostatic potential fitting family includes CHELPG, HLY, MK, and RESP. The deformation density family includes Hirshfeld and VDD. Stockholder partitioning methods include a diverse set that spans a wide variation in average charge transfer magnitudes: Hirshfeld, ACP, i-ACP, DDEC6, ISA, Hirshfeld-I, MBIS, and Becke. Although from a methodology perspective the stockholder partitioning methods form a class, their charge assignment results are diverse. For example, Hirshfeld NACs are highly correlated to VDD NACs (both are based on the deformation density) but not to the Hirshfeld-I NACs.^1^ From a statistical perspective, DDEC6 NACs were very highly (>0.95) correlated to MBIS, Hirshfeld-I, ISA, and IBO NACs for molecules,^1^ but the DDEC6 average charge transfer magnitude more closely resembled that of the electrostatic potential fitting group, IBO, and i-ACP than the average charge transfer magnitudes of MBIS, Hirshfeld-I, and ISA.

The high confluence ranking of DDEC6 cannot be solely attributed to either the presence of other stockholder partitioning methods in the dataset nor to the presence of electrostatic potential fitting methods in the dataset. Consider a pared down dataset in which all stockholder partitioning methods except DDEC6 and all electrostatic potential fitting methods are removed so that only ADCH, APT, CM5, DDEC6, EEQ, IBO, MBSMulliken, QTAIM, and VDD remain. As shown in Table 6, DDEC6 remains the top-ranked method in this pared down dataset.

**Table 6:** Rankings of nine charge assignment methods in a pared down dataset. The *S*_*α*_ and *Ω*(*α*, *ϕ*) ranking criteria always give the same order of methods

Rank	Method	*S*_*α*_	*Ω*(*α*, *ϕ*)	Method	*Ω*(*α*, MPC)	Method	Number (*Ω*_*αβ*_ > 0.8)	Method	Number (*Ω*_*αβ*_ > 0.9)
1	DDEC6	8.111	0.977	DDEC6	0.978	DDEC6	9	DDEC6	5
2	IBO	7.967	0.960	IBO	0.962	VDD	8	CM5	5
3	CM5	7.899	0.952	CM5	0.954	IBO	7	MBSMulliken	5
4	MBSMulliken	7.868	0.948	MBSMulliken	0.951	CM5	7	IBO	4
5	EEQ	7.856	0.946	EEQ	0.948	MBSMulliken	7	EEQ	4
6	VDD	7.733	0.932	VDD	0.931	EEQ	7	QTAIM	2
7	ADCH	7.417	0.894	ADCH	0.896	ADCH	7	APT	2
8	QTAIM	7.046	0.849	QTAIM	0.843	APT	4	VDD	1
9	APT	7.013	0.845	APT	0.839	QTAIM	3	ADCH	1

### 3.4 An unambiguous scientific test of atomic population analysis methods

Confusion on whether it is possible to apply the scientific method to quantify properties of atoms in materials pertains to the issue of whether atom-in-material properties can be experimentally measured. While it is generally believed that NACs are not directly measurable experimentally, the situation is actually two-fold. For the vast majority of materials NACs are not directly measurable experimentally, but a few carefully chosen materials provide clear enough experimentally measured atomic population data for falsifiable scientific tests. It is obvious that atom-in-material properties for a completely isolated atom are experimentally measurable. For example, the NAC of a completely isolated Na^+^ ion could be definitively measured in an experiment to be +1. However, this is not helpful, because all atomic population analysis methods would yield the correct NAC in this case. The challenge is to come up with more interesting cases where the experimental result is unambiguous and some population analysis methods fail unambiguously. Here, I show that such situations do indeed occur. In other words, I show it is possible to unambiguously falsify some atomic population analysis methods using the scientific method. By unambiguously, I mean the conclusion is independent of opinions, interpretations, and perspectives.

As an example, consider the endohedral N@C_60_ system in which a N atom sits inside a C_60_ cage. Electron paramagnetic resonance (EPR) and electron nuclear double resonance (ENDOR) experiments showed the ground spin state is *S* = 3/2.^70^ (The ground spin state of an isolated N atom is also *S* = 3/2.) These spectra also show the N atom occupies a central position and interacts only weakly with the C_60_ cage.^70–73^ “… from the missing nuclear quadrupole interaction a symmetric on-centre equilibrium position of the nitrogen atom can be deduced, implying an isotropic g-matrix.”^74^ The interaction between the enclosed N atom and C_60_ cage is sufficiently weak that at room temperature the cage spins freely around the enclosed N atom leading to a spherically symmetric environment observed in the EPR and ENDOR experiments.^73^ How much spin density is transferred between the enclosed N atom and the C_60_ cage? “… because of the undetectable ^13^C hyperfine interaction, the admixture of fullerene molecular orbitals to the central atom wavefunction seems to be extremely small and, as a result, spin rotational interaction can also be neglected. (A ^13^C hyperfine interaction of the order of 0.05 mT corresponding to approximately 1.5 MHz is expected for a unit spin density on the C_60_ shell. The observed 50 kHz linewidth therefore puts an upper limit of 3% to the transferred spin density.)”^74^ In other words, the amount of spin transferred from the enclosed N atom to the C_60_ cage is small or negligible. How much net charge is transferred from the enclosed N atom to the C_60_ cage? “The UV/vis spectrum of N@C_60_ is indistinguishable within experimental error from that of C_60_, confirming negligible coupling between nitrogen in its atomic ground state and C_60_ cage molecular wave functions.”^75^ If the C_60_ cage in N@C_60_ carried a substantial net charge, this would have altered its UV-vis spectrum compared to isolated C_60_. Because the UV-vis spectrum was unaltered, net charge transfer from the enclosed N atom to the C_60_ cage is negligible or small in magnitude.

The [Eu@C_60_]^+^ system exhibits remarkably different behavior than N@C_60_. First, the Eu atom in Eu@C_60_ is markedly off-center.^76^ Second, there is strong interaction between the Eu atom and the C_60_ cage. In contrast to the UV-vis spectrum of N@C_60_ which was equivalent to the isolated C_60_ spectrum, the Eu@C_60_ UV-vis spectrum shows dramatic differences.^77^ Comparing the Eu L_III_-edge XANES spectra of Eu@C_60_ to reference compounds showed the Eu atom in Eu@C_60_ is in the +II oxidation state.^77^ This implies the seven 4f electrons comprising a half-filled subshell remain on the Eu atom,^77^ along with potentially part of the 6s electrons. The 4f electrons have a smaller average radius and are more tightly bound than the 6s electrons. “The [isolated] C_60_ host has only deeply held paired electrons.^78^ (Experiments show C_60_ has a first ionization energy of 6.4–7.9 eV, an electron affinity of approx. 2.6–2.8 eV, and a first optical transition of approx. 3.2 eV.^79–82^)”^15^ Therefore, electrons may be transferred from the Eu atom to the C_60_ cage, but would not be transferred from the C_60_ cage to the Eu atom. Together, these results show the Eu atom in [Eu@C_60_]^+^ should have a NAC between approximately 1 and 2 and an ASM between approximately 7 and 8.

Table 7 summarizes computed NACs for 20 methods having a complete basis set limit. ASMs are also listed for those methods that compute them. These calculations used the PBE/def2TZVPP optimized geometries and wavefunctions computed in Gaussian 16.^64^ The same software programs were used to compute the NACs of these systems as were used for the water molecule in Section 3.1. An extremely fine (0.04 bohr) grid was used for the QTAIM method. Default settings were used for all other methods. The Multiwfn defaults for CHELPG, MK, and RESP used vdW radii of 1.5 Å for C, 1.5 (MK and RESP) or 1.7 (CHELPG) for N, and 1.4554 for Eu. As recommended in the paper introducing the RESP method, a hyperbolic penalty function was used with two-stage fitting and constants of *a* = 0.0005 (stage 1), *a* = 0.001 (stage 2 on selected atoms), and *b* = 0.1 (both stages).^33^ For comparison, Table 7 also shows a one-stage RESP fitting using the strong constraint (*a* = 0.001, *b* = 0.1) on all atoms.

**Table 7:** Falsifiable scientific tests of 20 methods to assign NACs in molecular systems. The NAC and ASM of the central atom are listed for each method

Method	N@C_60_		[Eu@C60]^+^	
	NAC	ASM	NAC	ASM
ACP	−0.017	a	a	a
ADCH	0.126	2.720^b^	0.476	6.891^b^
APT	0.015	c	0.415	c
Becke	−0.056	2.900	−4.427	7.001
CHELPG	0.371	c	1.031	c
CM5	0.120	2.720^b^	1.016	6.891^b^
DDEC6	0.143	2.836	1.360	6.933
EQeq	−0.081	c	1.278	c
Hirshfeld	0.139	2.720	0.525	6.891
Hirshfeld-I	0.147	2.788	1.483	6.892
HLY	1050.40	c	199.86	c
i-ACP	−0.009	a	a	a
IBO	−0.013	2.987	d	d
ISA	−3.082	2.800	1.452	6.910
MBIS	0.157	2.821	e	e
MBSMulliken	−0.019	2.981	f	f
MK	11.986	c	0.926	c
QTAIM	0.014	2.888	2.691	6.932
RESP	9.116 [6.553]^g^	c	0.925 [0.925]^g^	c
VDD	0.198	2.906	0.339	6.931

a The ACP and i-ACP parameters are not yet defined for the element Eu. Although the ACP and i-ACP methods could yield ASMs, this is not yet available in the software.

b ASMs for the ADCH and CM5 methods are taken from the Hirshfeld partition.

c This method does not give ASMs.

d IBOView version 20150427 could not compute IBO populations for atoms using a RECP.

e The software used was not set up to compute MBIS populations for atoms using a RECP.

f MBSMulliken was not available for the Eu element in the Gaussian 16 program.

g Two-stage fitting without brackets. One-stage fitting in brackets. See text for RESP penalty function parameter values.

Several observations are:

(1) Because the nuclear charge of N is +7, its maximum possible NAC of +7 would be achieved if all electrons were removed from this atom. The two-stage RESP NAC of 9.116 for the N atom clearly shows this method assigns a negative number of electrons (*i.e.*, −2.116 electrons) to this atom. The same problem occurred for the HLY and MK analysis of N in N@C_60_. Because the number of electrons cannot properly be negative, these methods are falsified for the N@C_60_ system. The one-stage RESP NACs using the strong constraint gave a NAC of 6.553 for the N atom which is much too high even though it is slightly below the atomic number of 7 for N.

(2) The ISA method gave a NAC of −3.082 for the N atom in N@C_60_, which is much too large in magnitude. Therefore, ISA is falsified for this material.

(3) The HLY NAC of 199.86 for the Eu atom in [Eu@C_60_]^+^ is unphysically high. The maximum physically possible NAC for an Eu atom would be +63 if all of its electrons were removed. Hence, HLY is falsified for the [Eu@C_60_]^+^ system.

(4) The Becke method gives a NAC of −4.427 for the Eu atom in [Eu@C_60_]^+^. This is chemically unreasonable, because electrons in the C_60_ cage are tightly bound and would not be transferred to the Eu atom. Therefore, the Becke method for computing NACs is falsified for the [Eu@C_60_]^+^ system.

(5) The QTAIM NAC of 2.691 for Eu in [Eu@C_60_]^+^ leaves 9 (valence electrons for neutral Eu) – 2.691 = 6.309 valence electrons which are too few to explain the QTAIM ASM of 6.932 for Eu in this material. (If all of these remaining valence electrons were spin polarized they would produce an ASM of 6.309.) Hence, this QTAIM NAC is a bit too high in magnitude.

These results show some atomic population analysis methods are falsified for these materials using the scientific method. This does not necessarily imply those particular methods will not work for other materials, but it indicates those methods may not be reliable across diverse material types.

The observant reader will notice N@C_60_ contains a ‘buried’ nitrogen atom. For comparison, the water molecule studied in Table 2 does not contain any buried atoms. A buried atom is any atom whose shortest distance to the material's van der Waals surface exceeds that atom's van der Waals radius. Materials with buried atoms are plentiful: all liquids, all solids (except one- and two-atom thick materials), and some gasses and plasmas contain buried atoms. Some molecules containing five or more atoms have buried atoms. As indicated in Table 1 and described in prior literature, the CHELPG, HLY, ISA, and MK methods fail for many materials with buried atoms.^23,33,52^ The RESP method was developed with the intention to fix this problem,^33^ but results for N@C_60_ presented here show the RESP method is not reliable for fixing this problem in some materials. Changing the form or strength of the RESP constraints could potentially address this problem, but this example clearly demonstrates the extreme challenge associated with trying to find a RESP constraint that works well across diverse materials. Notably, it is not as easy as just making the constraints stronger or weaker, because a RESP constraint that is too strong for one material (or for one part of a material) may be too weak for another material (or for a different part of the same material).

Although the N@C_60_ material contains a buried atom, the presence or absence of buried atoms played no role in the decision to select this material as a benchmark system. N@C_60_ was chosen as a benchmark material, because to the best of the author's knowledge published experimental spectroscopic results have characterized its net atomic charges and atomic spin moments more accurately and definitely than for any other known material containing unpaired electron spins and at least two different atom types. As described earlier in this section, these experimental data show unambiguously that there is small or negligible charge and spin transfer from the N atom to the C_60_ cage and the system's ground state is a spin quartet.

## 4. Seven confluence principles

The word confluence means a coming together, joining, or merging. In the statistical context of this paper, confluence denotes a joining together or merging of statistical characteristics. Two statistical characteristics that are normally thought to be distinct may actually merge to become a single characteristic. Also, various physical or statistical properties may be simultaneously highly correlated to a single quantitative descriptor.

**Fig. 3:**
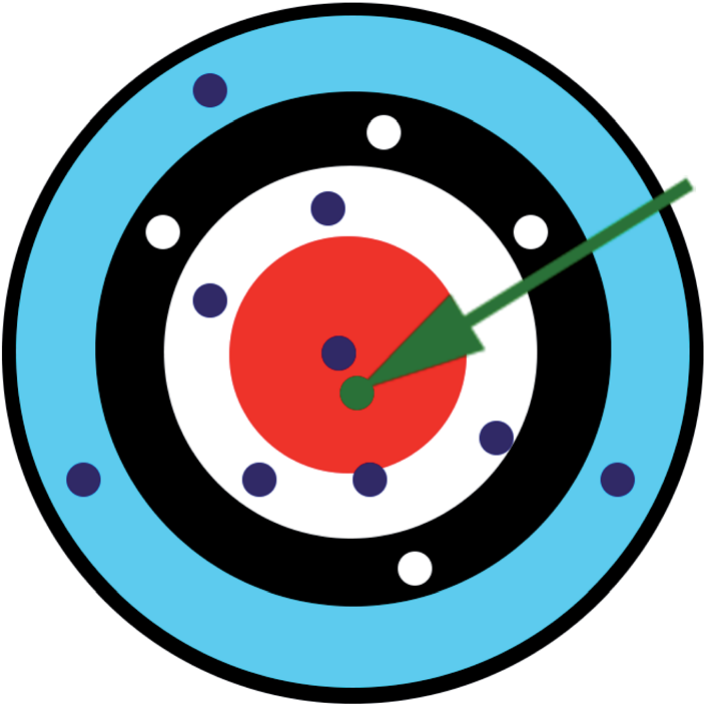
In a group of darts aimed at any target, the centrally located dart never lands farthest from the target. If the group of darts follows a spherically symmetric distribution, then a centrally located dart lands closer to the target than at least ∼50% of the darts. In other words, the centrally located dart performs average or better for diverse targets. This centrally located dart exhibits confluence properties including high correlation to the other individual darts and to the main principal component of the dart group.

An analogy is useful. As illustrated in Fig. 3, consider a group of darts aimed at some target. The dart located in the center of the group never lands the farthest from any conceivable target. This centrally located dart exhibits confluence properties including high correlation to the other individual darts and to the main principal component of the dart group. If the group of darts follows a spherically symmetric distribution, then a centrally located dart lands closer to the target than at least ∼50% of the darts. In other words, the centrally located dart performs average or better for diverse targets. Other individual darts may land closer to the bullseye for specific targets, but the centrally located dart is best positioned for general-purpose use across diverse targets.

Confluence is the missing link that shows how to define quantitative descriptors that are not directly experimentally observable (at least in most cases) to achieve high correlations to a host of related physical properties. In this article, we consider the task of assigning properties to atoms in materials. Atoms are the conceptual foundation of chemistry; however, many properties of individual atoms in materials are not directly observable experimentally for most materials. For example, the partial charge (*i.e.*, NAC) of an atom in a material is not a direct experimental observable for most materials. Nevertheless, the concept of charged atoms (*i.e.*, anions and cations) has been crucial to understanding the chemistry of many materials. By using confluence, a NAC descriptor can be constructed that exhibits good correlations to a host of chemical properties related to the partial charges of atoms in materials.

The remainder of this section precisely defines confluence and seven associated confluence principles.

### 4.1 Definition

A quantitative descriptor is defined as confluent among a group of positively correlated quantitative descriptors if this quantitative descriptor has sufficiently high correlation to the group's average standardized variable *ϕ*. The precise threshold for “sufficiently high” must be (arbitrarily) chosen. Example: as shown in Table 4, the correlation *Ω*(DDEC6, *ϕ*) = 0.985 can be considered “sufficiently high” to label DDEC6 as a confluent descriptor for NAC methods.

### 4.2 Confluence principle #1

For a group of positively correlated quantitative descriptors, the descriptor with the highest correlation to the group's average standardized variable (*Ω*(*α*, *ϕ*)) also has the highest sum of correlations to the individual group members (*S*_*α*_). Proof: eqn (31) shows *S*_*α*_ = *S*_*ϕ*_*Ω*(*α*, *ϕ*). Because *S*_*ϕ*_ is the same for all group members, the group member with highest *Ω*(*α*, *ϕ*) also has highest *S*_*α*_. Example: as shown in Table 4, the highest values correspond to *S*_DDEC6_ = 18.204 and *Ω*(DDEC6, *ϕ*) = 0.985, which are related by *S*_*α*_/*Ω*(*α*, *ϕ*) = *S*_*ϕ*_ = 18.4806. Implication: the centrally located dart exhibits not only the strongest correlation to the group's average position, but also the highest sum of correlations to all positions of the individual darts in the group.

### 4.3 Confluence principle #2

PCA of the correlation matrix for a group of quantitative descriptors yields coefficients for the *k*^th^ principal component (PCk) that are directly proportional to each descriptor's correlation to this PC. Proof: the correlation between standardized variable *α̂* and the *k*^th^ principal component directly expands to give39
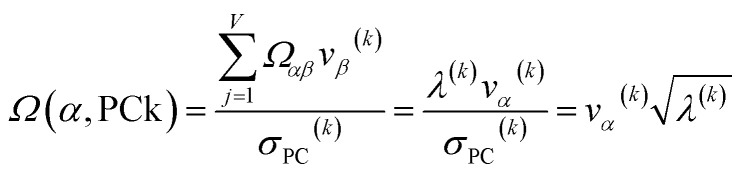
where *v*_*α*_^(*k*)^ is the coefficient for *α̂* in the *k*^th^ eigenvector of the correlation matrix, *λ*^(*k*)^ is the corresponding eigenvalue, and 
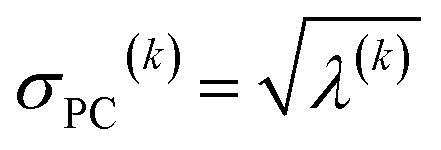
 (eqn (21)) is the standard deviation of PCk across the datapoints. Example: as shown in Table 3, the MK method had a coefficient of 0.229 in the MPC, and the MPC eigenvalue = 17.158. Thus, correlation of MK to the MPC = 0.229 × sqrt(17.158) = 0.949, as verified in Table 4. Implication: after performing PCA of the correlation matrix, the ranking of variables according to their coefficients in the MPC is identical to the ranking of variables according to their correlation to the MPC.

### 4.4 Confluence principle #3

For a group of positively correlated quantitative descriptors, a quantitative descriptor's correlation to the group's average standardized variable *ϕ* is similar (though not necessarily equal) to the same descriptor's correlation to the correlation MPC. Proof: see Section 7.3. Example: in Table 4, the largest difference magnitude between a single descriptor variable's correlation to *ϕ* and MPC is 0.007. Implication: ranking variables according to (i) *Ω*(*α*, *ϕ*) (equivalent to *S*_*α*_ ranking) or (ii) *Ω*(*α*, MPC) (equivalent to MPC coefficient ranking) yields similar (not necessarily equal) results.

### 4.5 Confluence principle #4

Among a group of positively correlated quantitative descriptors, the quantitative descriptor exhibiting confluence to the group's average standardized variable *ϕ* has predictive advantages across a broad range of target applications. Explanation: here, the term “predictive advantages” refers to the fact that a centrally located dart will not land farthest from any related target (see Fig. 3). If the darts are approximately uniformly distributed over a spherical region, then the center dart lands closer to any target than at least ∼50% of the darts. This analogy extends to quantitative descriptors where a centrally located descriptor is a descriptor that is highly correlated to *ϕ*. Example: as an example, DDEC6 NACs (which have high correlation to *ϕ*) give good performance across both chemical properties and electrostatic properties of molecules.

### 4.6 Confluence principle #5

If a group of positively correlated quantitative descriptors contains two confluent descriptors *α* and *β*, then descriptors *α* and *β* are somewhat highly correlated to each other. Proof: using standardized variables *α̂*, *β̂*, and *φ̂*, the correlations are proportional to the dot products over the sample data points: *Ω*_*αβ*_ = ·(*α̂, β̂*)/*M*, *Ω*(*α*, *ϕ*) = ·(*α̂, φ̂*)/*M*, and *Ω*(*β*, *ϕ*) = ·(*β̂, φ̂*)/*M*, where dot product has the following definition40
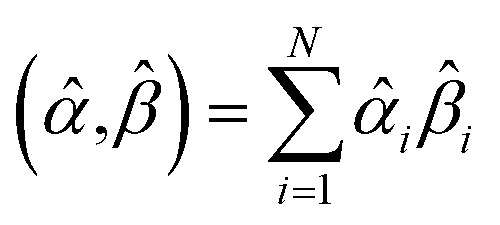


Because the variables are standardized, ·(*α̂, α̂*) = ·(*β̂, β̂*) = ·(*φ̂, φ̂*) = *M*. Because of this normalization, ·(*α̂, φ̂*) ≈ *M* if and only if *α̂* is approximately parallel to *φ̂*. If descriptors *α* and *β* are both confluent, this means *Ω*(*α*, *ϕ*) ≈ *Ω*(*β*, *ϕ*) ≈ 1, which can only occur if ·(*α̂, φ̂*) ≈ ·(*β̂, φ̂*)≈ *M*. In other words, *α* and *β* must both be approximately parallel to *ϕ*, which can only occur if they are also approximately parallel to each other. This therefore implies that ·(*α̂, β̂*) ≈ *M*, and thus that *Ω*_*αβ*_ ≈ 1. Example: comparing Fig. 2 to Table 4, the 15 descriptors having correlation > 0.9 to the DDEC6 NACs (the most confluent descriptor among the 20 NAC methods) were exactly the same 15 descriptors having highest correlation to *ϕ*. The Spearman rank correlation between correlation to DDEC6 and correlation to *ϕ* was 0.90 across the 20 methods, which reveals similar (but not completely identical) rankings according to correlation to DDEC6 NACs and correlation to *ϕ*. Implication: this principle shows arbitrariness in designing descriptors is dramatically reduced when those descriptors are designed to be confluent. Specifically, two different descriptors, each designed to be confluent across the same descriptor group, will be highly correlated to each other and thus not arbitrarily valued.

### 4.7 Confluence principle #6

If quantitative descriptor A is optimized to be confluent among a group of target physical properties, this same quantitative descriptor is expected to be confluent among a group of quantitative descriptors that are individually highly correlated to individual physical properties in this group. Explanation: suppose there are a group of physical properties designated P1, P2, P3, *etc.* that are experimentally measured across a sample population. Suppose further the quantitative descriptor A has been optimized to give high positive correlations between descriptor A and each individual physical property P1, P2, P3, *etc.* across this sample population. In other words, the correlation between descriptor A and property P1 is high across this sample population. The correlation between descriptor A and property P2 is also high across this sample population, and so forth for properties P3, *etc.* Now suppose there is another quantitative descriptor B1 that is optimized to give high positive correlation to physical property P1 across this sample population, but not necessarily high correlation between B1 and physical property P2 or P3 across this sample population. Now suppose there is another quantitative descriptor B2 that is optimized to give high positive correlation to physical property P2 across this sample population, but not necessarily high correlation between B2 and physical property P1 or P3 across this sample population. Now suppose there is another quantitative descriptor B3 that is optimized to give high positive correlation to physical property P3 across this sample population, but not necessarily high correlation between B3 and physical property P1 or P2 across this sample population. Likewise descriptors B4, B5, *etc.* are highly correlated to physical properties P4, P5, *etc.*, respectively. Since descriptor A is confluent among related physical properties P1, P2, P3, *etc.*, then it will also be confluent among a group of descriptors B1, B2, B3, *etc.* that are optimized to be highly correlated to physical properties P1, P2, P3, *etc.* Proof: because the standard deviation of any standardized variable across the sample population equals one, high positive correlations between descriptor A and properties P1, P2, *etc.* can only occur if41
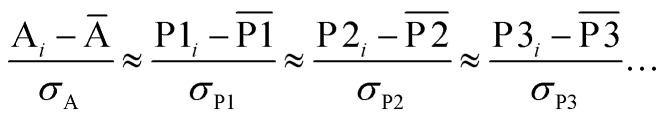
for the vast majority of data points in the sample, where the overbar represents the average across the sample population and A_*i*_, P1_*i*_, *etc.* represent the descriptor and property values for the *i*^th^ datapoint in the sample population. Since descriptor B1 is highly positively correlated to property P1, it follows that42
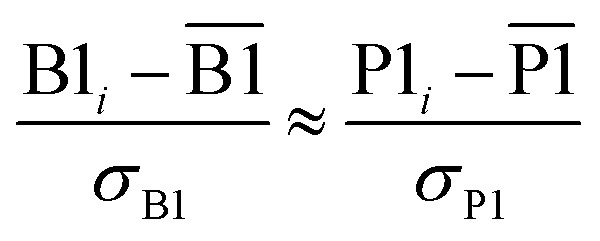
for the vast majority of data points in the sample. Similarly, a high positive correlation between descriptor B2 and property P2 means that43
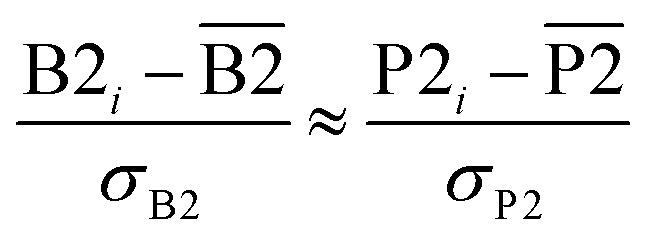
for the vast majority of data points in the sample. Combining eqn (41)–(43) gives44
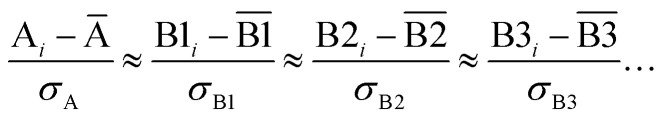
for typical data points in the sample, which completes the proof. Implication: some NAC methods (*e.g.*, ACP, ADCH, CM5, i-ACP) were optimized to reproduce molecular dipole moments. Others were optimized to reproduce the electrostatic potential surrounding the molecule (*e.g.*, CHELPG, HLY, MK, RESP). Others were optimized to maximize the similarity to quantum-mechanically computed reference atom densities (*e.g.*, Hirshfeld, Hirshfeld-I) or orbitals (*e.g.*, IBO). Others were optimized to reproduce constrained (*e.g.*, MBIS) or unconstrained (*e.g.*, ISA) spherically averaged AIM distributions. Others were optimized to reproduce electronegativity trends (*e.g.*, EEQ) or number of electrons in the volume dominated by each atom (*e.g.*, QTAIM). There are two different approaches to achieve high correlations across the majority of these descriptors. In approach II, we optimize a quantitative descriptor to be strongly correlated to a collection of many various related quantitative descriptors. In other words, we could optimize a NAC method to give NACs that are strongly correlated to the NACs produced by many various NAC methods. In approach I, we optimize a quantitative descriptor to strongly correlate to many various physical properties (MEP, molecular dipole moments, element electronegativities, *etc.*). For example, optimizing NACs to reproduce a variety of physical and chemical properties. Regardless of whether approach I or approach II is chosen, the end result is similar: the resulting descriptor will be confluent across this descriptor group and the related physical and chemical properties. Example: the DDEC6 NACs were designed to be confluent across various physical and chemical properties; they were not developed with the goal of giving strong correlations to other NAC assignment methods.^15^ Nevertheless, they consequently developed strong correlations to other NAC assignment methods as demonstrated by the data in Table 4 and Fig. 2.

### 4.8 Confluence principle #7

The MPC of the correlation matrix is the solution to a confluent optimization, where the MPC is a normalized linear combination of members of a descriptor group: (a) the MPC maximizes correlation variance across the dataset and (b) the MPC maximizes the sum of squared correlations to individual members of the descriptor group. Either criterion (a) or (b) could be enforced leading to identical MPC. Proof: see Section 7.4. Implications: MPC has a high combination of correlations to the individual members of the descriptor group. Whereas the average standardized variable *ϕ* maximizes the sum of correlations to the individual group members, the MPC maximizes the sum of squared correlations to the individual group members. This means that both *ϕ* and MPC are maximally correlated to the individual group members, and therefore likely strongly correlated to each other. Example: in agreement with the *ϕ* and MPC optimization criteria, Table 8 shows *ϕ* exhibits higher summed correlation compared to MPC, while MPC exhibits higher summed squared correlation compared to *ϕ*. Because of confluence, the differences are tiny. Expanding the correlation between *ϕ* and MPC gives45
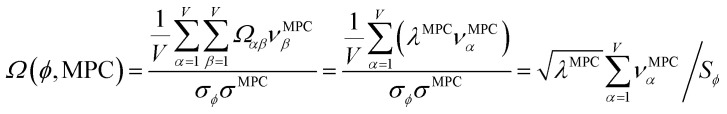


**Table 8:** Summed correlations and summed squared correlations between *ϕ* or MPC and the NAC methods

	Summed correlations	Summed squared correlations
*ϕ*	18.48063	17.15555
MPC	18.47951	17.15756

For the NAC methods, *λ*^MPC^ = 17.15756, *S*_*ϕ*_ = 18.48063, and 
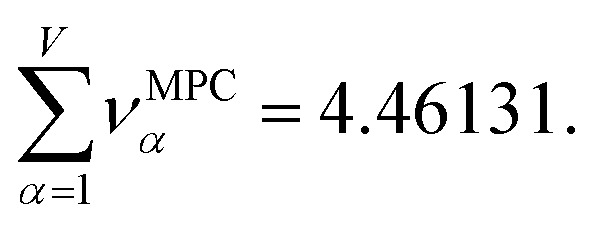
 Inserting these values into eqn (45) gives *Ω*(*ϕ*, MPC) = 0.99994, which clearly indicates an almost perfect correlation between *ϕ* and MPC for the NAC methods. Clearly, *Ω*(*ϕ*, MPC) must be less than one, but it is extremely close to one for the group of NAC methods.

## 5. How these confluence principles work together with other key principles and the scientific method to make assigning atom-in-material properties non-arbitrary

In spite of the importance of atoms in materials to all chemical sciences, there historically existed severe dysfunction when it comes to quantifying properties of atoms in materials. The scattershot performance of early atomic population analysis methods contributed to this confusion. Of the 26 methods considered in this work, the oldest are Mulliken (introduced in 1955 (ref. 30)), QTAIM (introduced in 1972 (ref. 32)), and Hirshfeld (introduced in 1977 (ref. 21)). The correlation between QTAIM and Hirshfeld is low (*Ω*_QTAIM–Hirshfeld_ = 0.762). Moreover, the Hirshfeld and QTAIM average charge transfer magnitudes are the extreme smallest and largest, respectively, of all 26 methods considered for the molecular systems. The Mulliken NACs have no complete basis set limit. Consequently, a concept emerged in the early days that NACs are an extremely ill-determined ‘arbitrary’ concept. This idea of NAC arbitrariness was further encouraged by many poorly performing methods introduced in subsequent decades, often without a clear understanding of the limitations of various approaches. For example, the Bickelhaupt, MBSBickelhaupt, Stout–Politzer, and Ros–Schuit methods lack rotational invariance; they produce different results when the entire molecule is rotated with respect to the coordinate axes. Because scalar properties like NACs are not vectors or tensors, their values should be independent of coordinate system orientation. Therefore, NAC methods lacking rotational invariance are unphysical. The Lowdin method, which was not included in Cho *et al.*'s dataset, was another early method that exhibits strong basis set dependence. While the original Lowdin method lacks rotational invariance,^83^ the subsequent Davidson–Lowdin^84^ method is rotationally invariant but still lacks a complete basis set limit. Some methods also had convergence problems: either failing to converge in some cases or converging to non-unique solutions. Some good methods were also introduced along the way.

Methods for assigning atom-in-material properties should preferably work across an extremely wide range of material types. Arguments for a specialized atomic population analysis method that is specifically optimized for a narrow material class are intrinsically weak. An atomic population analysis method that is specifically optimized to describe one material class (and not other material classes) will be unable to describe systems containing that one material class together with other material types. For example, one person may claim to have developed a new atomic population analysis method that is specifically optimized to describe ionic liquids and not other materials, while another person may claim to have developed a different atomic population analysis method that is specifically optimized to describe metal–organic frameworks and not other materials. Neither of these methods are capable of describing the behavior of ionic liquids in metal organic frameworks, because they fail to simultaneously describe both material classes. As a second example, even though there are many different kinds of molecules, a charge assignment method that only works for molecules is quite limited, because it cannot even describe systems in which molecules react on solid surfaces. Since the number of possible chemical combinations is infinite, this requires a general-purpose atomic population analysis method that applies across an extremely wide range of material types.

While there exists some flexibility in constructing atomic population analysis methods, this flexibility should be constrained in several key ways:

### Criterion 1

Atom-in-material properties should be mathematically well-defined with a complete basis set limit and rotational invariance.^10^

### Criterion 2

The method for computing atom-in-material descriptors should be physically well-motivated and derivable from fundamental principles.^10,56,85,86^

### Criterion 3

If the value of an atom-in-material descriptor corresponds to functional minimization, this functional should be convex to ensure the minimum is unique.^15,53,85^ Moreover, nearly flat optimization landscapes should be avoided.^33,53^

### Criterion 4

For the reasons discussed above, methods for assigning atom-in-material properties should preferably work across an extremely wide range of material types containing both surface and buried atoms.

### Criterion 5

For carefully selected benchmark systems, the computed atom-in-material descriptor value should approximately match known reference values. For NACs and ASMs, the N@C_60_ system discussed above is one such example. Examples of known bond orders include the H_2_ (BO = 1), N_2_ (BO = 3), and O_2_ (BO = 2) molecules.

### Criterion 6

The method for computationally assigning atom-in-material properties should be compatible and consistent across various quantum chemistry methods. For example, it should give consistent results for different basis set types (*e.g.*, plane waves, Gaussian, *etc.*) as well as for methods having idempotent (*e.g.*, DFT, HF) and non-idempotent (*e.g.*, CCSD, CAS-SCF, SAC-CI, *etc.*) first-order density matrices.^10^ One way to achieve this is to make the assigned atom-in-material descriptors functionals of the electron density and spin magnetization density distributions.^56^

### Criterion 7

Each computed atom-in-material descriptor should be designed to achieve confluence across related properties. In other words, it should be strongly correlated to many related experimentally measured and theoretically computed physical and chemical properties.

### Criterion 8

An atomic population analysis method should preferably be capable of computing a whole suite of atom-in-material properties (net atomic charges, atomic spin moments, bond orders, spdfg populations, *etc.*) as opposed to assigning only one atom-in-material property.

### Criterion 9

The assigned values of atom-in-material properties should be chemically consistent. For example, the number of electrons assigned to an atom should be non-negative. Also, various atom-in-material descriptor values (*e.g.*, NACs, ASMs, bond orders, spdfg populations) should be non-contradictory (*i.e.*, approximately consistent with each other). For example, a hydrogen atom should not be assigned an ASM of 0.9 and a NAC of 0.75, because the former requires at least 0.9 electrons to reside on this atom while the latter requires 0.25 electrons to reside on this atom.

### Criterion 10

The assigned atom-in-material descriptors should have good transferability between similar chemical environments.^87,88^ Conformational transferability is especially important when parameterizing flexible force fields.^27,53,89^

### Criterion 11

An atomic population analysis method should have reasonable computational costs.^10^ (Note: the prior literature contains detailed computational cost studies for a small number of individual atomic population analysis methods, but no study has been published to date that systematically compares computational costs across a wide range of different atomic population analysis methods.^52,55,90–93^)

### Criterion 12

The atomic population analysis method should not require the manual adjustment of computational parameters for individual systems; it should work out of the box without requiring system-specific tweaking from a human.^85^

### Criterion 13

The assigned electron density partitions “should be localized around the atomic nucleus and should not have intricate structures far from their defining nuclear center. This requirement is usual necessary, albeit insufficient, for chemical transferability and conformational stability.”^85^

### Criterion 14

The assigned atom-in-material properties should properly reflect the material's symmetry.^10^

### Criterion 15

For atom-in-material descriptors in which the sum over atoms has a well-defined value, this value should be properly reproduced. For example, the NACs should sum to the unit cell's net charge,^10^ for collinear magnetism the ASMs should sum to the number of spin-up minus spin-down electrons in the unit cell, *etc.* Also, the local values of the electron density partitions should add up to the total electron density at each position in space (eqn (37)).

How does confluence specifically relate to assigning atom-in-material charges? NACs could be optimized to reproduce the electrostatic potential surrounding a molecule (*e.g.*, CHELPG, HLY, MK methods, *etc.*), to reproduce the molecular dipole moment (*e.g.* ADCH, *etc.*), to reproduce dipole moment derivatives (*i.e.*, APT), to correspond to virial compartments (*i.e.*, QTAIM), to match deformation densities (*i.e.*, Hirshfeld, VDD), to project onto a basis of atomic orbitals (*e.g.*, IBO, MBSMulliken, *etc.*), to maximize similarity between reference ions and assigned atom-in-material electron distributions (*e.g.* Hirshfeld-I, *etc.*), or to satisfy other criteria. But do these optimization criteria require different NAC methods? Could a NAC method be developed that simultaneously provides reasonably good correlations to most of these criteria?

Confluence is the concept of a centrally located method that is ideally positioned to give good correlations to a broad range of related physical properties. In the analogy of a group of darts aimed at targets, the central dart never lands farthest from any target. Confluence is a viable approach to constructing a truly general-purpose atomic population analysis method.

Confluence removes much of the arbitrariness associated with constructing an atomic population analysis method. It may appear arbitrary whether the NACs should be optimized to reproduce (a) the MEP, (b) the molecular dipole moment, or (c) the number of electrons in the local volume dominated by each atom-in-material, *etc.* However, much of this arbitrariness can be removed by optimizing NACs to achieve confluence across these various physical properties. We may conceivably construct at least two different atomic population analysis methods K and L which are each confluent across these target physical properties. According to confluence principle #5, the results of atomic population analysis methods K and L will be positively correlated to each other. Because each of the target properties (a) to (c) listed above are linear in the charge transfer magnitude, methods K and L must have similar charge transfer magnitudes to be confluent across these properties. Hence, the results of methods K and L must be approximately similar.

For example, the NAC for an oxygen atom in an optimized isolated water molecule is between approximately −0.6 and −0.85 for methods optimized to criteria (a) or (b). As shown in Table 2, the 4 MEP fitting methods (criterion (a)), CM5 and ADCH (criterion (b)), and DDEC6 (confluence across criteria (a) to (c)), Becke, MBSMulliken, i-ACP, IBO, and ISA NACs were within this range. The QTAIM (criterion (c)), VDD, Hirshfeld, EQeq, APT, ACP, MBIS, and Hirshfeld-I NACs were not within this range.

Manz and co-workers developed an extremely wide-ranging suite of atomic population analysis tools called the Standard Atoms in Materials Framework (SAMF): (i) ASMs for materials with collinear and non-collinear magnetism,^86^ (ii) bond orders,^56^ (iii) orbital bond order components that sum to the correct bond orders,^94^ (iv) atom-in-material polarizabilities, dispersion coefficients, and quantum Drude oscillator parameters,^95,96^ (v) various generations of charge partitioning schemes,^15,52–54^ (vi) many linear-scaling computational algorithms,^55,56,86,96^ and (vii) a complete library of charge-compensated reference ions for all charge states of chemical elements 1 to 109.^15,52,53,97^ This charge-compensated reference ion library and methods to compute ASMs, bond orders, bond order components, polarizabilities, dispersion coefficients, and quantum Drude oscillator parameters can in principle be used with multiple charge assignment methods. However, consistently high accuracy is obtained only when using a high-quality and extremely versatile charge partitioning method such as DDEC6 or similar.^15,54,56,95^ When these techniques are used with DDEC6 or potentially similar high-quality partitioning, all 15 criteria described above can be satisfied.

The DDEC6 method is strongly based on confluence. DDEC6 atomic population analysis was developed to achieve confluence across various observable chemical properties rather than to maximize its correlation to other atomic population analysis methods. The DDEC6 NACs are simultaneously optimized to give strong correlations to: (i) the electrostatic potential surrounding the material, (ii) the number of electrons in the local volume dominated by each atom-in-material, (iii) dipole moments, (iv) element electronegativities, and other properties.^15^ The DDEC ASMs are simultaneously optimized to resemble proportional spin partitioning and spherical averaging of the spin magnetization density vectors.^55,86^ The Manz bond orders, which often use DDEC6 charge partitioning, are based on the confluence of atom-in-material exchange propensities.^56^ The MCLF atom-in-material polarizabilities and dispersion coefficients (which often use DDEC6 charge partitioning) are based on m-scaling, conduction limit upper bound, and other scaling principles to achieve accurate results for both surface and buried atoms in diverse materials.^95,96^

According to confluence principle #6, this will naturally result in strong correlations between DDEC6 NACs and NACs computed by other methods. This is clearly demonstrated by the data in Table 4 and Fig. 2. According to confluence principle #4, this gives DDEC6 atomic population analysis predictive advantages across a wide range of target applications.

In summary, there is some flexibility in designing atomic population analysis approaches, but various approaches that produce a chemical descriptor strongly correlated to many related physical properties must also produce strong correlations in-between these different atomic population analysis approaches. In other words, there may be several paths to achieve similar descriptor values. This is why methods like DDEC6 and IBO that are based on entirely different approaches yield similar average charge transfer magnitudes and highly correlated NACs for small molecules. It is not the values themselves of atom-in-material properties, but various paths to achieve similar values that embodies most of the flexibility of constructing general-purpose atomic population analysis methods. Incorporating diverse material classes (*e.g.*, molecules, dense solids, porous solids, conductors, insulators, magnetic materials, multi-reference systems, *etc.*) and computational approaches (*e.g.*, DFT and various correlated wavefunction methods) can reveal which strategies are broadly applicable and which perform well only for specific material types. Methods that perform well only for limited material types should be replaced by more broadly applicable methods.

## 6. Conclusions

Assigning properties to atoms in materials is not arbitrary. It is theoretically possible to develop a method to assign NACs that simultaneously has average or better correlations to any and all physical and chemical properties related to atom-in-material charges. In other words, it is theoretically possible to develop a universally good method to assign NACs and other atom-in-material properties. This can theoretically be achieved by centrally locating the atomic population analysis method so that it exhibits strong correlations to other atomic population analysis methods. Among existing atomic population analysis methods, the DDEC6 method currently comes closest to this ideal.

Linear least-squares fitting is an extremely common technique. However, simple least squares fitting (SLSF) is not reversible: a SLSF of variable *y* to *x* yields a linear model that is not mathematically equivalent to a SLSF of variable *x* to *y*.^42^ A bivariate standardized reversible linear least squares fitting was introduced here that solves this problem and has four important properties: (i) it is a total least squares fit with Euclidean metric, (ii) it is an orthogonal distance regression, (iii) it is a PCA regression, and (iv) it has a universal model equation that requires no computerized calculations. Because of property (iv), it is called instant least squares fitting (ILSF). The ILSF universal linear model equation can be applied to any pair of positively correlated quantitative descriptors; however, it will achieve the best results when those two descriptors are approximately linearly correlated to each other.

As an example, this ILSF was used to compute average charge transfer magnitudes of 26 different methods to assign NACs across ∼2000 molecular systems. The Hirshfeld and VDD methods (which partition deformation densities) had the smallest average charge transfer magnitudes, while QTAIM (which assigns virial compartments) had the largest average charge transfer magnitude. NACs (*e.g.*, ACP, ADCH, CM5, i-ACP) intended to reproduce the molecule's dipole moment had smaller average charge transfer magnitudes than those NACs optimized to reproduce the electrostatic potential surrounding the molecule (*e.g.*, CHELPG, HLY, MK, RESP). The Bickelhaupt, DDEC6, IBO, and ISA methods gave average charge transfer magnitudes similar to the electrostatic potential fitting group.

The correlation matrix between 20 NAC methods having complete basis set limits was extensively analyzed. This correlation matrix had a main block comprised of 14 NAC methods with strong inter-correlations plus two small side blocks weakly connected to the main block. Principal components analysis showed the main (or first) principal component accounts for 17.16 variables' worth (85.8%) of the correlation in this group. Each of the other principal components accounted for less than one variable's worth of correlation apiece. The NAC methods were ranked according to how strongly correlated they are to all 20 NAC methods. The top (DDEC6), the bottom (Becke), the 2^nd^ (MBIS), the 8^th^ (RESP), the 9^th^ (MK), the 11^th^ (CM5), and the 15^th^ (Hirshfeld) ranked methods had consistent rankings across various ranking criteria. The DDEC6 method exhibited correlation >0.9 to 15 of 20 methods, and it had a summed correlation = 18.204. The Becke method exhibited *R* < 0.7 to all other NAC methods. The DDEC6 NACs had correlation *R* = 0.986 and 0.985 to the MPC and average standardized variable *ϕ*, respectively. The MBIS, ISA, and Hirshfeld-I NACs also exhibited high correlations to these descriptors and other NAC methods.

Calculations in Section 3.3 showed the top ranking is relatively stable to the choice of which other charge assignment methods are included in the dataset. For example, the top-ranked method was unchanged when the number of different charge assignment methods was increased to 26 or decreased to 9.

Although NACs are not unambiguously measurable experimentally for most materials, N@C_60_ is an interesting benchmark case for which experimental spectroscopy results show negligible or small charge and spin transfer from the N atom to the C_60_ cage. Calculations in Section 3.4 for N@C_60_ showed that some charge assignment methods give unphysical results for this material while other charge assignment methods performed well. This example demonstrates that it is possible, at least in some cases, to falsifiably test atomic population analysis methods using the scientific method.

Seven confluence principles were derived that explain many connections between correlation properties. For example, NAC methods ranked similarly according to the sum of correlations to other methods (*S*_*α*_), correlation (*Ω*(*α*, *ϕ*)) to the average standardized variable *ϕ*, coefficient in the correlation main principal component (MPC), correlation to this MPC, and number of NAC methods to which a NAC method is strongly correlated (*e.g.*, *Ω*_*αβ*_ > 0.9). Many relations between these correlation properties were derived and proved.

A quantitative descriptor with strong correlations to many related descriptors has predictive advantages across multiple applications. This can be illustrated *via* an analogy to a group of darts aimed at a target. A dart near the center of the group lands closer to the bullseye than at least ∼50% of the darts in all cases, irrespective of the target. This confluence should be used to construct general-purpose atomic population analysis methods. A general-purpose atomic population analysis method should have NACs that are confluent across properties related to atomic charges, bond orders that are confluent across properties related to bond orders, ASMs that are confluent across properties related to the atom-in-material ordering of unpaired electron spins, and so forth.

In addition to achieving confluence, a general-purpose atomic population analysis method should also satisfy many other criteria as described in Section 5. For example, it should give chemically accurate results across diverse material types, give consistent results across different quantum chemistry methods (*e.g.*, various basis sets and exchange-correlation theories), be capable of computing many different atom-in-material descriptors that are approximately chemically consistent with each other, have approximate transferability of descriptor values between similar chemical environments, be computationally efficient, not require manual tweaking for individual materials, have good convergence properties, and so forth.

Finally, the correlations reported in this article for ∼2000 main group molecules (which contain many surface atoms) should not be extrapolated to dense solids (which contain many buried atoms). It often occurs that two charge assignment methods give similar results for surface atoms but vastly different results for buried atoms.^33,52^ Future studies should consider more diverse material types. The most confluent method identified in this study (*i.e.*, DDEC6) was previously shown to perform well across an extremely broad range of material types: small organic and inorganic molecules, dense solids, porous solids, nanostructured materials, large biomolecules, ionic liquids, polymers, organometallic complexes, heterogenous and homogenous catalysts, metal–organic frameworks, conductors, semi-conductors, and insulators, *etc.*^15,54–56,98,99^ Moreover, DDEC6 yields a wide range of atom-in-material properties: bond orders,^56^ net atomic charges and atomic multipoles,^15,54^ atomic spin moments,^55,86^ polarizabilities and dispersion coefficients and quantum Drude oscillator parameters (when used in conjunction with the MCLF method^95,96^), electron cloud parameters,^15,100^ and bond order components.^94^ DDEC6 has been used to construct flexible force fields for various materials.^101–112^

## 7. Appendix: mathematical proofs

### 7.1 Proof that total least squares and orthogonal distance regression yield the same reversible linear model

Consider a linear model of the form

46 *z*_*i*_ ≈ *ζw*_*i*_ + *η*

Because the error measure in eqn (15) is reversible, the model's predicted values are related to the measured values *via* the equations

47 *z*^pred^_*i*_ = *ζw*^measured^_*i*_ + *η*

48 *w*^pred^_*i*_ = *z*^measured^_*i*_/*ζ* − *η*/*ζ*

Using the error measure of approach 1,

49 Δ*w*_*i*_ = *w*^pred^_*i*_ − *w*^measured^_*i*_ = −Δ*z*_*i*_/*ζ*

Δ*z*_*i*_ = *z*^pred^_*i*_ − *z*^measured^_*i*_50
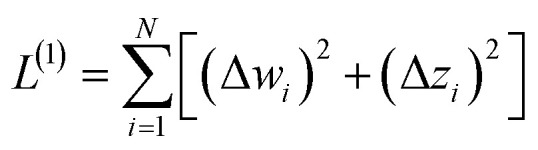
which rearranges to give51
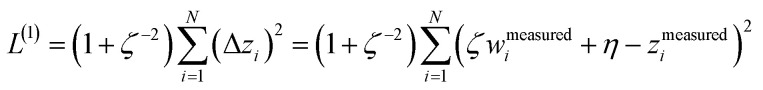
The minimum occurs when



52

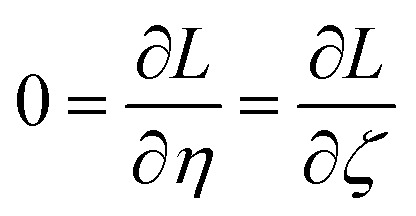

First,53
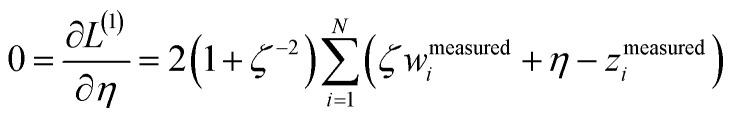
simplifies to

54 0 = 2(1 + *ζ*^−2^)*N*(*ζw*_avg_ + *η* − *z*_avg_)

By definition (see eqn (6) and (7)), *z*_avg_ = *w*_avg_ = 0. Because *w* and *z* are real-valued and positively correlated, *ζ* is real-valued and non-zero. Also, *N* ≥ 2. Accordingly, eqn (54) simplifies to *η* = 0. Putting *η* = 0 into eqn (51) and simplifying gives

55 *L*^(1)^ = (1 + *ζ*^−2^)*M*(*ζ*^2^ − 2*ζΩ*_*wz*_ + 1)

where the sums have been evaluated in terms of the correlation matrix elements (eqn (2) and (5)). Second,56
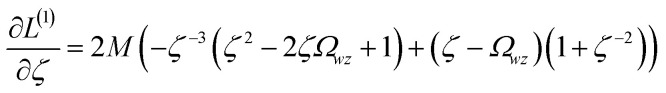
which simplifies to57
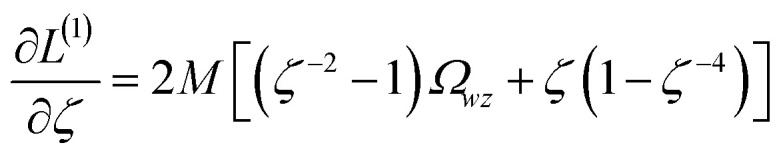
which has only two real-valued roots: *ζ* = −1 and *ζ* = 1. The condition *ζ*(1 + *ζ*^−2^) = *Ω*_*wz*_ would also yield ∂*L*/∂*ζ* = 0, but cannot be satisfied for any real value of *ζ* for 0 < *Ω*_*wz*_ ≤ 1. Inserting these into eqn (55) yields

58 *L*^(1)^(*ζ* = −1) = 4*M*(1 + *Ω*_*wz*_)

59 *L*^(1)^(*ζ* = 1)=4*M*(1 − *Ω*_*wz*_)

*ζ* = 1 is the global minimum solution, because *w* and *z* are positively correlated by construction (*i.e.*, *Ω*_*wz*_ > 0).

Next, I show the same solution results from orthogonal distance regression of the standardized variables (approach 2). As shown in Fig. 1, the perpendicular distance is related to distances Δ*w* and Δ*z* that were considered in the total least squares fitting. Specifically, the area of the blue triangle in Fig. 1 is given by area = ½base × height = ½(Δ*w*)(Δ*z*) = ½*ht*. Hence,60
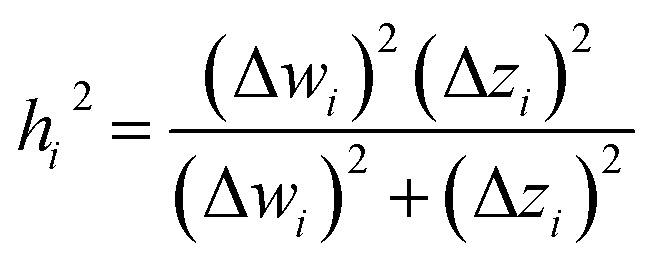
Substituting eqn (49) into (60) and simplifying gives61
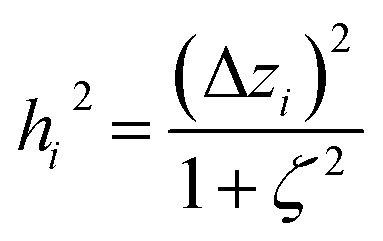
The orthogonal regression minimizes the sum of squared error (SSE)62
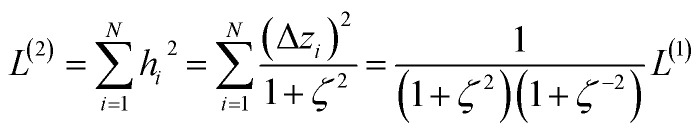
Hence63
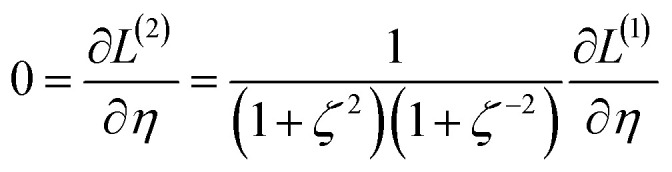


has the same solution *η* = 0 as above. Expanding64
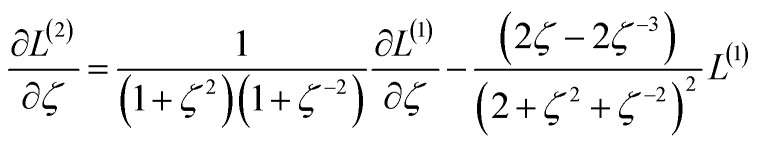
reveals ∂*L*^(2)^/∂*ζ* has exactly the same *ζ* = −1 and *ζ* = 1 roots as ∂*L*^(1)^/∂*ζ*. For these two roots, combining eqn (58), (59) and (62) yield

65 *L*^(2)^(*ζ* = −1) = *M*(1 + *Ω*_*wz*_)

66 *L*^(2)^(*ζ* = 1) = *M*(1 − *Ω*_*wz*_)

Hence, approach 1 (total least squares with Euclidean metric) and approach 2 (orthogonal distance regression) of the standardized variables produce exactly the same global minimum solution

67 (*ζ*, *η*)=(1, 0)

The quality of the fit can be quantified by −1 the sum of squared perpendicular errors divided by the sum of squared deviations of one variable from its average value:68
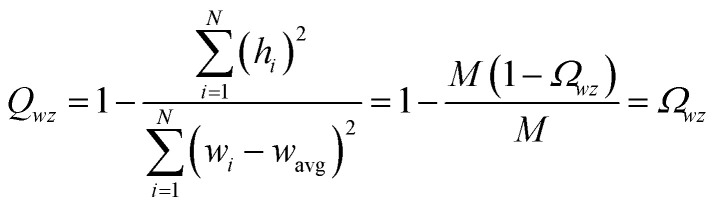
Hence, the fit quality equals the correlation. Because the variables are standardized, the same result occurs if *z* is used in place of *w* in the denominator of eqn (68).

### 7.2 Proof that *ϕ* maximizes summed correlations to the variables {*α̂*}

I now prove that *ϕ* is the descriptor that maximizes *S*_*τ*_ for any conceivable descriptor *τ* that is a linear combination of the standardized variables in a positively correlated descriptor group:69
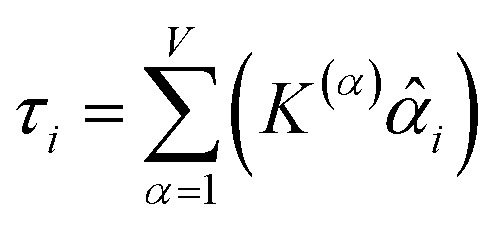


The standard deviation is70
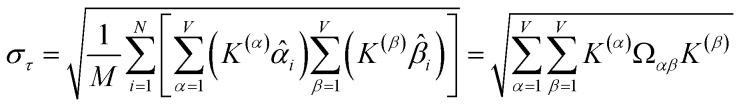


The objective function to be maximized expands as71
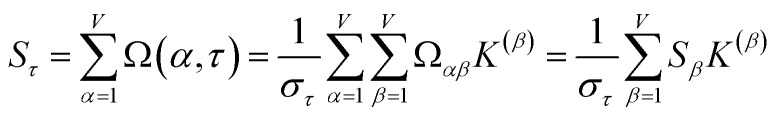
This is maximized when72
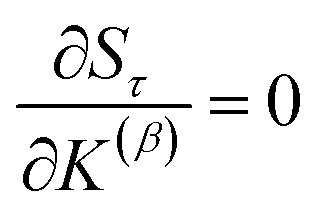
Differentiating eqn (71) gives73
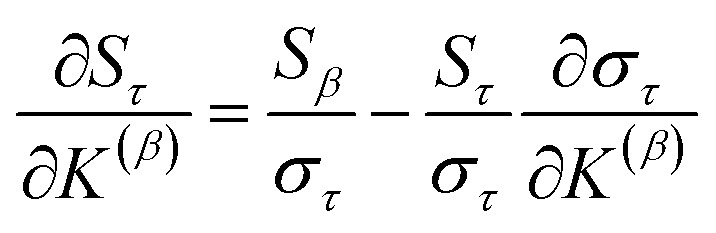
Differentiating eqn (70) gives74
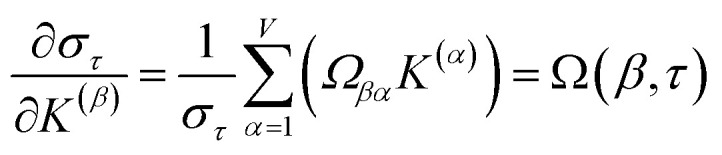
Inserting eqn (74) into eqn (73) and setting equal to zero gives75
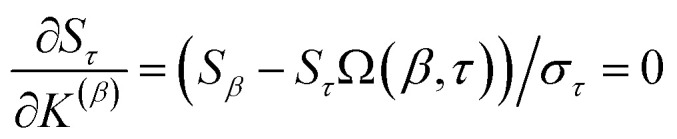
Examining eqn (31), eqn (75) is clearly satisfied for *τ* = *ϕ*, which proves that *ϕ* has the maximum possible summed correlations to the variables {*α̂*}.

### 7.3 Proof that a descriptor's correlation to *ϕ* and MPC are similar within a positively correlated descriptor group

Eqn (35) shows coefficients of the correlation MPC are approximately proportional to *S*_*α*_. The covariance between correlation MPC and standardized variable *α̂* is thus76
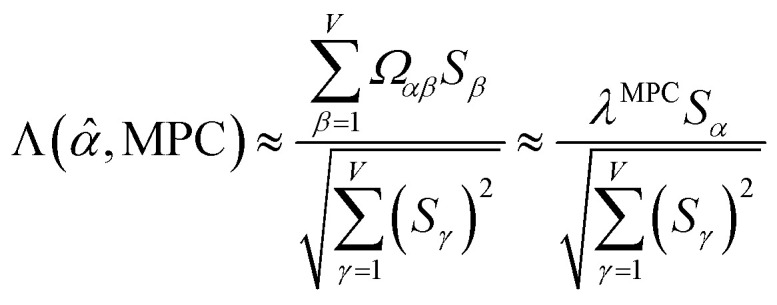
Dividing by 
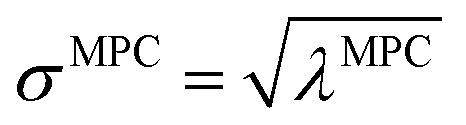
 gives the correlation:77
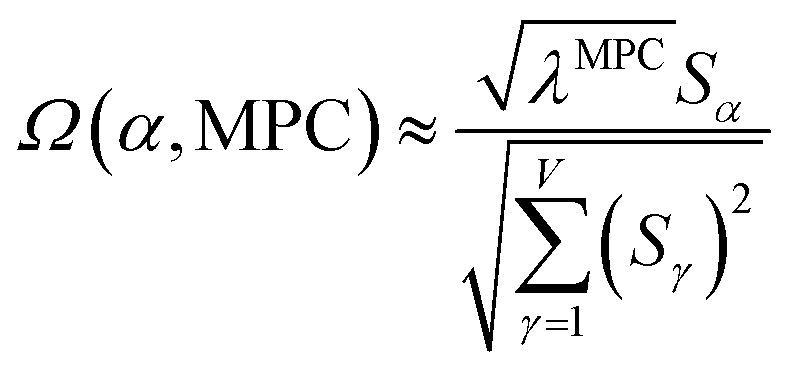


Eqn (31) shows *Ω*(*α*, *ϕ*) = *S*_*α*_/*S*_*ϕ*_. Comparing eqn (77) to eqn (31) proves *Ω*(*α*, MPC) is approximately proportional to *Ω*(*α*, *ϕ*). Furthermore, the power-law method to determine the largest eigenvalue (see eqn (32)) implies that78
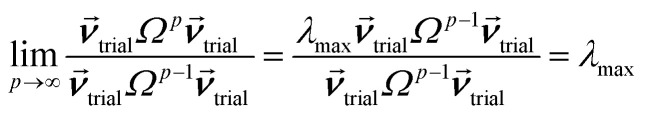


Using *p* = 2 and ***ν⃗***_trial_ = 1⃑ (*i.e.*, a vector filled with ones), gives79
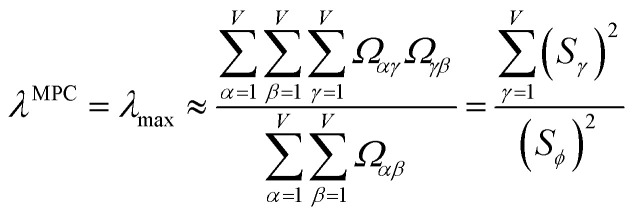
Inserting eqn (79) into eqn (77) and comparing to eqn (31) gives the final result

80 *Ω*(*α*, MPC) ≈ *Ω*(*α*, *ϕ*)

### 7.4 Proof the MPC maximizes the sum of squared correlations to individual members of a descriptor group

This section proves the following: the MPC of the correlation matrix is the solution to a confluent optimization, where the MPC is a normalized linear combination of members of a descriptor group: (a) the MPC maximizes variance across the dataset and (b) the MPC maximizes the sum of squared correlations to individual members of the descriptor group. Either criterion (a) or (b) could be enforced leading to identical MPC.

Enforcing the normalization constraint, the MPC variance is81
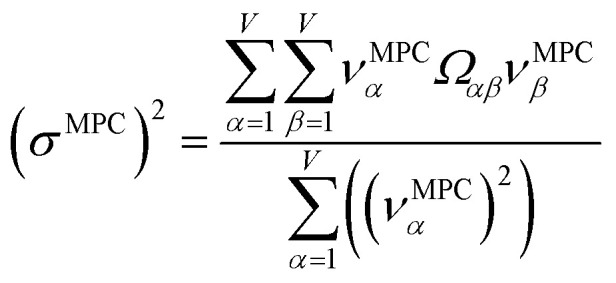


Following criterion (a), the variance is maximized by82
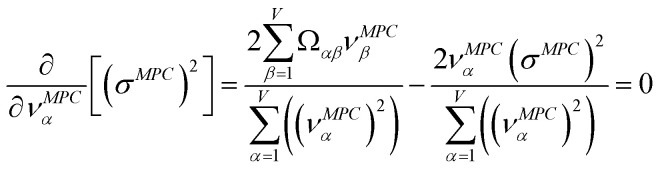
which is manifestly the eigenstate equation defining correlation MPC. The quantity maximized by criterion (b) is83
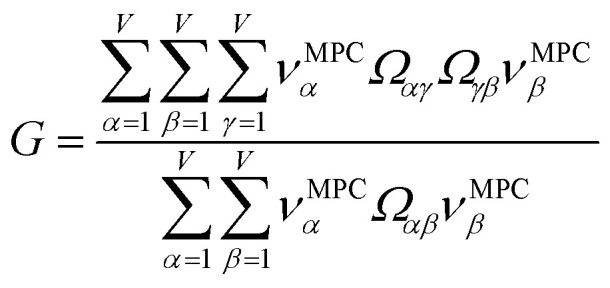


The derivative expands as84
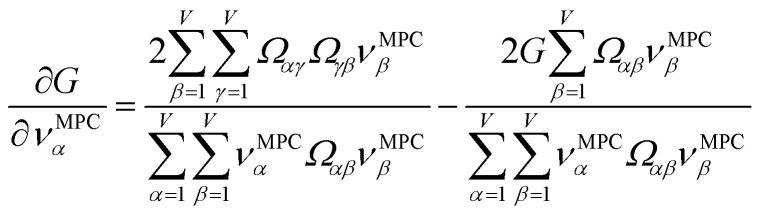


When *ν*^MPC^_*β*_ is an eigenvector of *Ω*_*γβ*_, this derivative simplifies to85
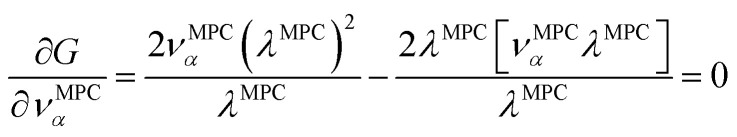


This maximizes *G* due to the derivative being zero. Therefore, criterion (b) has the same solution as criterion (a).

## Conflicts of interest

There are no conflicts of interest to declare.

## Supplementary Material
